# Coincident maps of changing land cover, land use, and forest condition in the United States, 1985-present

**DOI:** 10.1038/s41597-026-06743-0

**Published:** 2026-03-03

**Authors:** Ian W. Housman, Sean P. Healey, Joshua Heyer, Elizabeth Hardwick, Zhiqiang Yang, Jennifer Ross, Kevin Megown

**Affiliations:** 1 RedCastle Resources Inc, On-site contractor, U.S. Department of Agriculture Forest Service, Field Services & Innovation Center-Geospatial Office, Salt Lake City, USA; 2 U.S. Department of Agriculture Forest Service, Rocky Mountain Research Station, Riverdale, USA; 3 U.S. Department of Agriculture Forest Service, Field Services & Innovation Center-Geospatial Office, Salt Lake City, USA

**Keywords:** Ecological modelling, Environmental impact, Forest ecology, Scientific data, Environmental health

## Abstract

Maps of land cover class are more common, and generally more accurate, than maps of land use because “use” implies management intent that may not be directly sensible by earth-observing satellites. However, many monitoring frameworks related to sustainability require land use and land cover to be explicitly differentiated. This is particularly true for forests, where natural and human-caused dynamics in tree cover often occur independently of long-term land use changes that signal deforestation. We used an extensive multi-temporal, multi-variate sample of reference points across the United States to calibrate and validate 30 m mapped time series (1985–present) of land cover, land use, and vegetation condition change. These maps comprise the Landscape Change Monitoring System (LCMS) and are served through: an interactive, open-access app; Google Earth Engine; image services; and the FSGeodata Clearinghouse. Here, we provide methods, validation metrics, and a usage example highlighting the value of differentiating use from cover in the context of model-assisted estimation of forest area using U.S. Department of Agriculture, Forest Service inventory data.

## Background & Summary

Advances in data systems, data policies, classification methods, and reference data collection have led to the recent availability of several medium- to high-resolution maps of land cover class across large areas, including the entirety of the United States^1–6^.

The nomenclature used by these new products does not always strictly preserve the distinction between land cover and land use. Cover categories refer to vegetative, built, or physical features, whereas use categories indicate human decisions about purpose. Class definitions in these maps generally address land cover. Focus on surface elements is understandable, as those elements often have distinct properties when sensed remotely, but omission of land use can lead to incomplete or even misleading conclusions related to ecosystem sustainability.

For instance, a loss of tree cover in an area remaining forestland (a land use) will have significantly different impacts on ecosystem functions (e.g., carbon storage) than a loss of tree cover in an area where a change in land use prevents regrowth of lost trees. In fact, some efforts to create deforestation-free supply chains mandate elimination of timber harvests that result in land use conversion^7^. Differentiating land cover from land use dynamics will be vital in the monitoring that supports such initiatives. Land use is also key to understanding how, for example, a housing development with growing street trees might contribute to wildlife habitat in ways different from an area where similar in-growth of trees corresponds instead to forest establishment. Tracking only treed land cover classes in such residential areas might also lead to dubious conclusions about shrinking cities.

Additionally, many vegetation cover changes do not lead to conversion of land cover or land use class but are nevertheless ecologically important (e.g., thinning, insect and disease-related mortality, low-severity fire). Therefore, a third product, independent of land use and land cover class taxonomies, is needed to allow for tracking of vegetation disturbance and regrowth.

The Landscape Change Monitoring System (LCMS) is a remote sensing-based system produced by the United States Department of Agriculture Forest Service (Forest Service) for mapping: (1) vegetation cover Change (loss or gain); (2) Land Cover class; and (3) Land Use class. Maps produced by LCMS extend as an annual series from 1985 to the most recently completed growing year (this paper reflects methods, data, and validation results from the 1985–2023 LCMS output—v2023.9). LCMS is intended to provide a consistent monitoring method for applications including, but not limited to monitoring of: sensitive habitats, post-disturbance recovery, broad-scale vegetation cover change, and land cover/use conversion.

LCMS uses historical imagery from the Landsat and Copernicus Sentinel-2 (Sentinel-2) platforms, which support monitoring many types of ecological change^8^. Two time series processing algorithms—Landsat-based detection of Trends in Disturbance and Recovery (LandTrendr)^9,10^ and Continuous Change Detection and Classification (CCDC)^11^—are applied to Landsat and Sentinel-2 imagery prior to classification for the three product lines: Change, Land Cover, and Land Use (henceforth capitalized when referring to the LCMS product and lower case for the generic use of the term). Fitting a time series function to imagery across years has been demonstrated to both improve classification accuracy and stabilize annual classification in a way that reduces misleading class changes through time^12,13^.

Maps of land cover and land use condition or change, such as those derived from remotely sensed data and reported here, are often used as ancillary data supporting more precise estimates of forest characteristics from a designed sample of reference data. For example, Auch *et al*.^14^ used land cover and land use change maps from the Land Change Monitoring, Assessment, and Projection (LCMAP)^1^ product along with a statistical sample of multi-temporal reference data to estimate change among land cover classes in the United States. Olofsson *et al*.^15^, likewise outlined broadly applied methods using land change maps for sample poststratification. LCMS was co-developed with the Forest Service’s Forest Inventory and Analysis Program (FIA), which maintains a regularly-visited random sample of over 325,000 field plots across all U.S. lands^16^. Emerging tools support small-area estimation methods that leverage maps as ancillary data to increase precision of FIA’s plot-based estimates^17,18^. FIA measures land use independently of land cover to more directly monitor the type of real-world landscape dynamics referenced above, and the conservation of this distinction in the LCMS product suite may enhance support of FIA small-area initiatives.

This paper documents production methods, validation results, and delivery options for the LCMS Land Cover, Land Use, and vegetation Change products. An example of LCMS supporting FIA small-area estimation is presented under *Usage Notes*.

## Methods

**Fig. 1:**
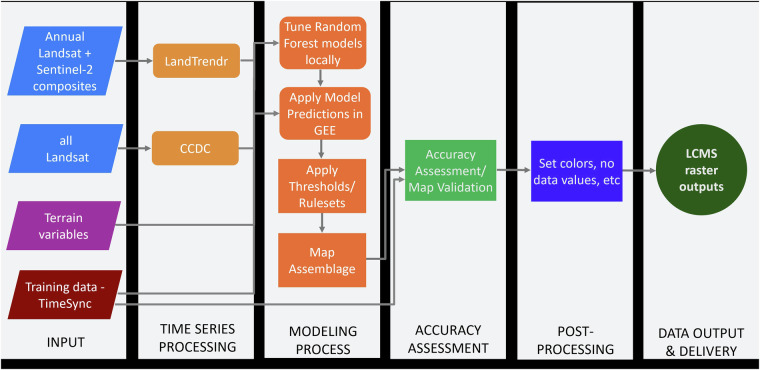
LCMS Workflow Flowchart. Overview of the Landscape Change Monitoring System (LCMS) workflow for producing Change, Land Cover, and Land Use datasets. The modeling process, accuracy assessment, post-processing, and data output and delivery steps are all conducted individually for producing Change, Land Cover, and Land Use outputs for each study area.

The LCMS production workflow for the Change, Land Cover, and Land Use products followed standard machine learning-based map production steps. This includes preparing model training and predictor data, model tuning, model prediction, map assembly, map accuracy assessment, post-processing, and data delivery (Fig. 1). For model training data (described below), we used a disproportionate stratified random sample design to generate locations for a training dataset collected using the TimeSync tool^19^. We created LCMS’ predictor data using Landsat and Sentinel-2 data in LandTrendr^9,10^ and Landsat in CCDC^11^ time series processing algorithms. We then used the Python package Scikit-Learn^20^ random forest^21^ for hyperparameter tuning. Next, we used the selected hyperparameters in the Google Earth Engine (GEE) instance of random forest to predict model outputs and assemble the map output. We then assessed map accuracy using a ten-fold cross validation method. Final steps were post-processing to format LCMS images, generate metadata, and set up LCMS data delivery and visualization platforms.

### Study areas

The 2023 LCMS (v2023.9, issued in 2024) mapping extent encompassed the conterminous United States (CONUS), southeastern Alaska (SEAK), Puerto Rico and U.S. Virgin Islands (PRUSVI), and the 8 major islands of Hawaii (HI). Interior Alaska is planned for inclusion in the 2024 LCMS release, scheduled for 2025 (v2024.10).

### Computing platforms

LCMS relies on Google Earth Engine^22^, accessed via an enterprise agreement between the Forest Service and Google, to handle all remote sensing raster data acquisition and processing. For sample design, predictor variable selection, and model validation, LCMS employs Scikit-Learn^20^ on local compute infrastructure.

### Model calibration data

Supervised statistical models require a set of calibration data (the dependent variable or training data), together with predictor variables (independent variables) to train the model. Once trained, the model is then applied to the predictor data (inference). This section details the procedures used to select and assign attributes to LCMS calibration data locations.

#### Model calibration data sample design

The main objective of our sample design was to effectively capture national-scale variability across vegetation cover change, land cover, and land use categories. Through pilot studies conducted across the U.S., we found that several important classes—such as vegetation cover loss and impervious land cover—occur relatively infrequently on the landscape. Our initial use of a simple random sample^23^ proved inadequate for representing these rare classes. To address this limitation, we adopted a disproportionate stratified random sample design, guided by recommendations from Olofsson *et al*.^14^. Specifically, “*The recommended allocation of sample size to the strata defined by the map classes is to increase the sample size for the rarer classes making the sample size per stratum more equitable than what would result from proportional allocation, but not pushing to the point of equal allocation.”* Following this approach, we stratified the landscape using available land cover/use and vegetation cover loss data, focusing on rare classes that were either central to LCMS applications and/or showed elevated model error in pilot studies:

- CONUS and SEAK: NLCD 2016 release^24^ 2016 mapped year land cover/use, LandTrendr-derived^9,10^ vegetation cover loss from 1984–2019, and the U.S. Geologic Survey State Geologic Map Compilation (SGMC)^25^
- HI: National Oceanic and Atmospheric Administration (NOAA) Coastal Change Analysis Program (C-CAP) 2010 Big Island^26^ land cover/use and LandTrendr-derived^9,10^ vegetation cover loss from 1990–2021
- PRUSVI: Helmer *et al*.^27^ land cover/use

We created the LandTrendr vegetation cover loss stratum from a 1984–2019 LandTrendr run for CONUS and SEAK and 1990–2021 for Hawaii, classifying any pixel that had any segment where NBR decreased by more than −0.2 as loss. Remaining pixels were classified as stable. This loss layer was used as a stand-alone stratum in Hawaii, where any loss pixel was part of its own stratum. In CONUS, loss was used in conjunction with Forest Service Regions and NLCD classes to create different strata for different tree types over the Eastern and Western U.S. The Western U.S. was defined as Forest Service Regions 1–6, while the Eastern U.S. was defined as Forest Service Regions 8 and 9. For SEAK, loss that fell within NLCD tree classes was used.

While our initial stratification addressed the lessons we learned in our pilot studies, our first CONUS-wide release (v2020.5) presented some new challenges:

- Commission of volcanic rock as Developed Land Use and Water Land Cover over the Sonoran and Mojave Deserts
- Commission of coastal wetlands as Developed Land Use over Southern Texas: these should be Non-Forest Wetland or Other Land Use classes
- Commission of Rangeland as Developed and Forest Land Use across areas of Southern Texas in areas of oil and gas extraction.

Starting in 2021, we introduced separate strata called Volcanic Rocks, South Texas Coastal Wetlands, and South Texas Oil and Gas to address these issues. For the Volcanic Rocks stratum, we began by drawing a rough polygon around the areas of Developed Land Use commission and another for Water Land Cover commission. Pixels were included as Volcanic Rocks if they:

- Were classified as Developed Land Use most often from 1985–2020 in the v2020.5 release of LCMS, had a value greater than 2 in the 2016 Global Human Settlement Layer (GHSL)^28^ built layer, and were classified as *Igneous, Volcanic* in the SGMC^25^, within the hand-drawn polygon.
- Were classified as Water Land Cover most often from 1985–2020 in the v2020.5 release of LCMS, had a slope > 0 degrees using the National Elevation Dataset^29^, and were classified as *Igneous, Volcanic* in the SGMC, within the hand-drawn polygon

For the Southern Texas strata, we first drew a rough polygon around the areas of Developed and Forest Land Use commission and another for Non-Forest Wetland Land Use. Pixels were included as Southern Texas Oil and Gas if they:

- Were classified as Developed Land Use most often from 1985–2020 in the v2020.5 release of LCMS, and had a value greater than 2 in the 2016 Global Human Settlement Layer (GHSL)^27^ built layer

Pixels were included as Southern Texas Coastal Wetlands if they:

- Were classified as Developed Land Use most often from 1985–2020 in the v2020.5 release of LCMS and were any of NLCD 2016 Barren Land (31), Deciduous Forest (41), Evergreen Forest (42), Scrub/Shrub (52), Grassland/Herbaceous (71), Woody Wetlands (90), or Emergent Herbaceous Wetlands (95).

The following are the strata we used, along with their respective source data:

- CONUS
  - Developed: NLCD 2016 Developed Open Space (21), Developed Low Intensity (22), Developed Medium Intensity (23), or Developed High Intensity (24)
  - Water: NLCD 2016 Open Water (11)
  - Snow/Ice: NLCD 2016 Perennial Ice/Snow (12)
  - Barren: NLCD 2016 Barren Land (31)
  - Agriculture: NLCD 2016 Cultivated Crops (82)
  - Herbaceous Wetlands: NLCD 2016 Emergent Herbaceous Wetlands (95)
  - Shrub/herb: NLCD 2016 Shrub/Scrub (52), Grassland/Herbaceous (71), or Pasture/Hay (81)
  - Evergreen Loss: NLCD 2016 Evergreen Forest (42) and LandTrendr Loss
  - Evergreen Stable: NLCD 2016 Evergreen Forest (42), and LandTrendr Stable
  - Deciduous West Loss: NLCD 2016 Deciduous Forest (41) or Mixed Forest (43), Forest Service Regions 1–6, and LandTrendr Loss
  - Deciduous West Stable: NLCD 2016 Deciduous Forest (41) or Mixed Forest (43), Forest Service Regions 1–6, and LandTrendr Stable
  - Deciduous East Loss: NLCD 2016 Deciduous Forest (41) or Mixed Forest (43), Forest Service Regions 8 and 9, and LandTrendr Loss
  - Deciduous East Stable: NLCD 2016 Deciduous Forest (41) or Mixed Forest (43), Forest Service Regions 8 and 9, and LandTrendr Stable
  - Volcanic Rocks: See above for detailed explanation
  - South Texas Coastal Wetlands: See above for detailed explanation
  - South Texas Oil and Gas: See above for detailed explanation
- SEAK
  - Developed: NLCD 2016 Developed Open Space (21), Developed Low Intensity (22), Developed Medium Intensity (23), or Developed High Intensity (24)
  - Water: NLCD 2016 Open Water (11)
  - Snow/Ice: NLCD 2016 Perennial Ice/Snow (12)
  - Barren: NLCD 2016 Barren Land (31)
  - Herbaceous and Woody Wetlands: NLCD 2016 Woody Wetlands (90) or Emergent Herbaceous Wetlands (95)
  - Dwarf Shrub/Herbaceous: NLCD 2016 Dwarf Scrub (51), Grassland/Herbaceous (71), Sedge/Herbaceous (72), Lichens (73), Moss (74), Pasture/Hay (81), or Cultivated Crops (82)
  - Tall Shrub Stable: NLCD 2016 Shrub/Scrub (52) and LandTrendr Stable
  - Tree Stable: NLCD 2016 Deciduous Forest (41), Evergreen Forest (42), or Mixed Forest (43) and LandTrendr Stable
  - Tree/Tall Shrub Loss: NLCD 2016 Deciduous Forest (41), Evergreen Forest (42), Mixed Forest (43), or Shrub/Scrub (52) and LandTrendr Loss
- PRUSVI
  - Developed: Helmer *et al*.^27^ High-Medium Density Urban (1) or Low-Medium Density Urban (2)
  - Water: Helmer *et al*. Background/water (0) or Water – Permanent (28)
  - Barren: Helmer *et al*. Salt or Mud Flats (20), Quarries (25), Coastal Sand and Rock (26), or Bare Soil (including bulldozed land) (27)
  - Agriculture: Helmer *et al*. Herbaceous Agriculture - Cultivated Lands (3) or Active Sun Coffee and Mixed Woody Agriculture (4)
  - Non-Forested Wetland: Helmer *et al*. Emergent Wetlands Including Seasonally Flooded Pasture (19)
  - Forested Wetland: Helmer *et al*. Mangrove (21), Seasonally Flooded Savannahs and Woodlands (22), Pterocarpus Swamp (23), or Tidally Flooded Evergreen Dwarf-Shrubland and Forb Vegetation (24)
  - Rangeland: Helmer *et al*. Pasture, Hay or Inactive Agriculture (e.g. abandoned sugar cane) (5), Pasture, Hay or other Grassy Areas (e.g. soccer fields) (6), or Drought Deciduous Open Woodland (7)
  - Evergreen: Helmer *et al*. Seasonal Evergreen and Semi-Deciduous Forest on Karst (13), Seasonal Evergreen and Evergreen Forest (14), Seasonal Evergreen Forest with Coconut Palm (15), Evergreen and Seasonal Evergreen Forest on Karst (16), or Evergreen Forest on Serpentine (17)
  - Deciduous: Helmer *et al*. Drought Deciduous Dense Woodland (8), Semi-Deciduous and Drought Deciduous Forest on Alluvium and Non-Carbonate Substrates (10), Semi-Deciduous and Drought Deciduous Forest on Karst (includes semi-evergreen forest) (11), or Drought Deciduous, Semi-deciduous and Seasonal Evergreen Forest on Serpentine (12)
  - Cloud Forest: Helmer *et al*. Elfin, Sierra Palm, Transitional and Tall Cloud Forest (18)
  - Coastal Mixed Forest: Helmer *et al*. Deciduous, Evergreen Coastal and Mixed Forest or Shrubland with Succulents (9)
- HI
  - Developed: C-CAP 2010 Impervious Surfaces (2) or Open Spaces Developed (2)
  - Water: C-CAP 2010 Water (21)
  - Barren: C-CAP 2010 Unconsolidated Shore (19) or Bare Land (20)
  - Agriculture: C-CAP 2010 Cultivated Land (6)
  - Wetland: C-CAP 2010 Palustrine Forested Wetland (13), Palustrine Scrub/Shrub Wetland (14), Palustrine Emergent Wetland (15), Estuarine Forest Wetland (16), Estuarine Scrub/Shrub Wetland (17), or Estuarine Emergent (18)
  - Rangeland: C-CAP 2010 Pasture/Hay (7) or Grassland (8)
  - Forest: C-CAP 2010 Evergreen Forest (10)
  - Scrub shrub: C-CAP 2010 Scrub/Shrub (12)
  - Loss: LandTrendr Loss

**Fig. 2:**
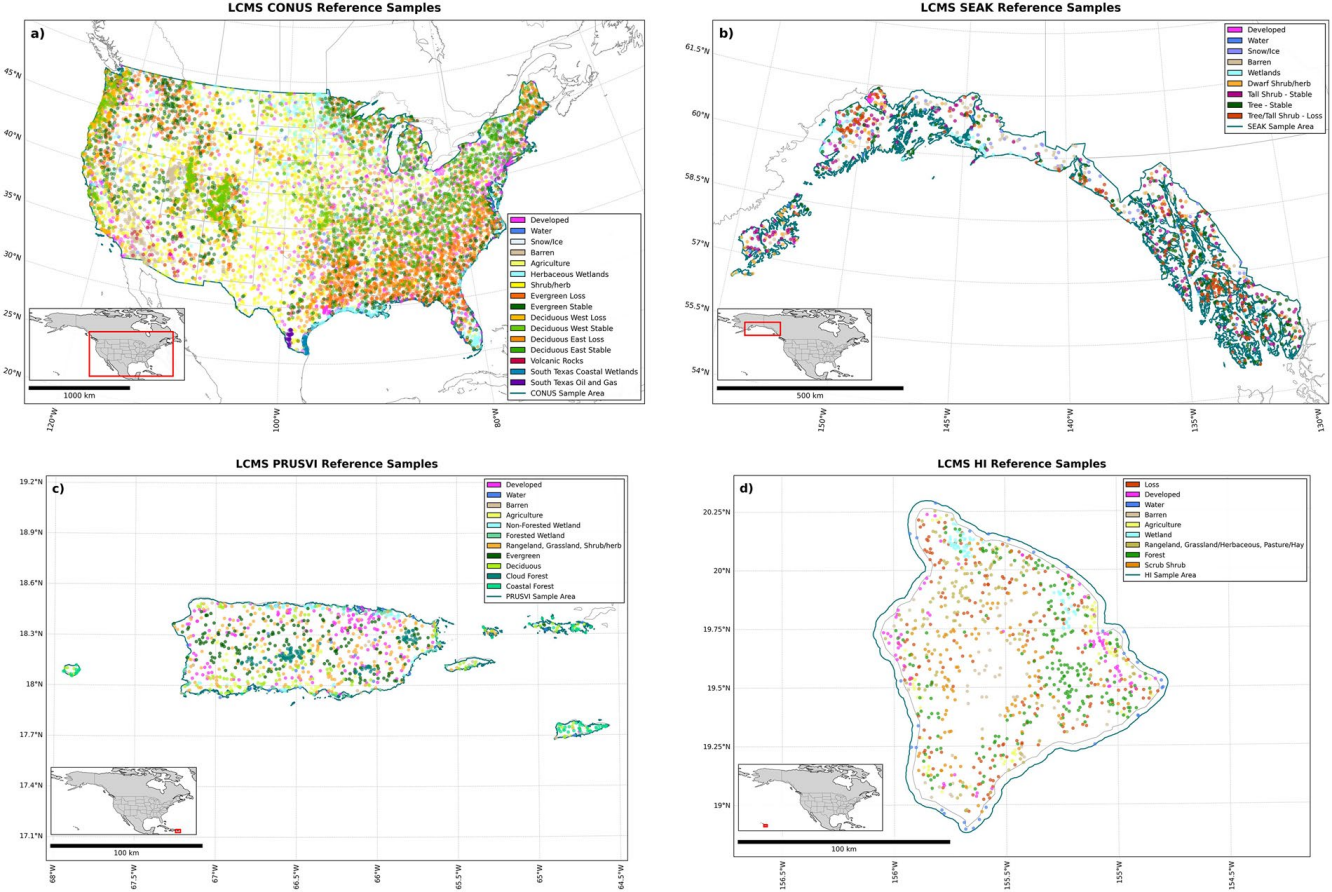
LCMS Reference Sample Maps. Maps of LCMS reference sample locations and respective strata for CONUS (a, top left), SEAK (b, top right), PRUSVI (c, bottom left), and HI (d, bottom right). Sample strata are shown with various colors to help illustrate the spatial distribution of the reference samples’ strata.

The final sample size consisted of 10,067 for CONUS, 929 for SEAK, 1,100 for PRUSVI, and 1,000 for HI (Fig. 2). The allocation process began with a distribution midway between equal and proportional. Maximum values were set at 1,000 for CONUS and 200 for SEAK, PRUSVI, and HI per stratum. The remaining samples were then proportionally and recursively allocated. Fixed sample sizes were established for certain classes: 30 for snow/ice (CONUS and SEAK only), 200 for water in CONUS, and 30 for water in SEAK, PRUSVI, and HI, reflecting their uniform variability. For PRUSVI, a fixed sample size of 30 was also assigned to barren. Table 1 presents the final sample counts, proportion, and weight by stratum for CONUS, SEAK, PRUSVI, and HI.

**Table 1:** Final sample counts, proportion of the total area (population), and corresponding strata weight for each stratum for the calibration samples for all study areas.

	Stratum	Sample Number	Proportion (%)	Weight		Stratum	Sample Number	Proportion (%)	Weight
CONUS	Developed	999	5.47	1.81	PRUSVI	Developed	157	14.14	1.01
	Water	200	1.72	1.15		Water	30	1.10	2.47
	Snow/Ice	30	0.01	45.02		Barren	30	1.07	2.54
	Barren	578	1.05	5.49		Agriculture	76	3.36	2.05
	Agriculture	1,007	16.89	0.59		Non-Forested Wetland	76	0.69	9.95
	Herbaceous Wetlands	659	1.44	4.56		Forested Wetland	57	0.91	5.72
	Shrub/Herb	1,010	43.50	0.23		Rangeland	200	34.32	0.53
	Evergreen Loss	1,280	4.37	2.91		Evergreen	200	32.43	0.56
	Evergreen Stable	988	11.99	0.82		Deciduous	114	8.44	1.23
	Deciduous West Loss	709	0.20	35.43		Cloud Forest	100	2.52	3.60
	Deciduous West Stable	533	0.81	6.55		Coastal Mixed Forest	60	1.00	5.47
	Deciduous East Loss	984	1.69	5.77		**TOTAL:**	1,100		
	Deciduous East Stable	990	10.79	0.91	HI	Developed	80	0.58	13.73
	Volcanic Rocks	34	0.02	20.89		Water	30	13.15	0.23
	S. Texas Coastal Wetlands	33	0.01	23.49		Barren	50	20.54	0.24
	S. Texas Oil & Gas	33	0.04	7.96		Agriculture	70	0.91	7.69
	**TOTAL:**	10,067				Wetland	70	2.49	2.81
SEAK	Developed	30	0.36	8.89		Rangeland	150	12.52	1.20
	Water	30	2.80	1.15		Forest	230	18.78	1.22
	Snow/Ice	30	22.37	0.14		Scrub shrub	120	12.00	1.00
	Barren	80	10.95	0.79		Loss	200	19.02	1.05
	Herb. & Woody Wetlands	79	3.06	2.78		**TOTAL:**	1,000		
	Dwarf Shrub/Herb	87	4.78	1.96					
	Tall Shrub – Stable	167	20.14	0.89					
	Tree – Stable	207	33.50	0.67					
	Tree/Tall Shrub Loss	219	2.04	11.55					
	**TOTAL:**	929							

#### Calibration data collection

**Fig. 3:**
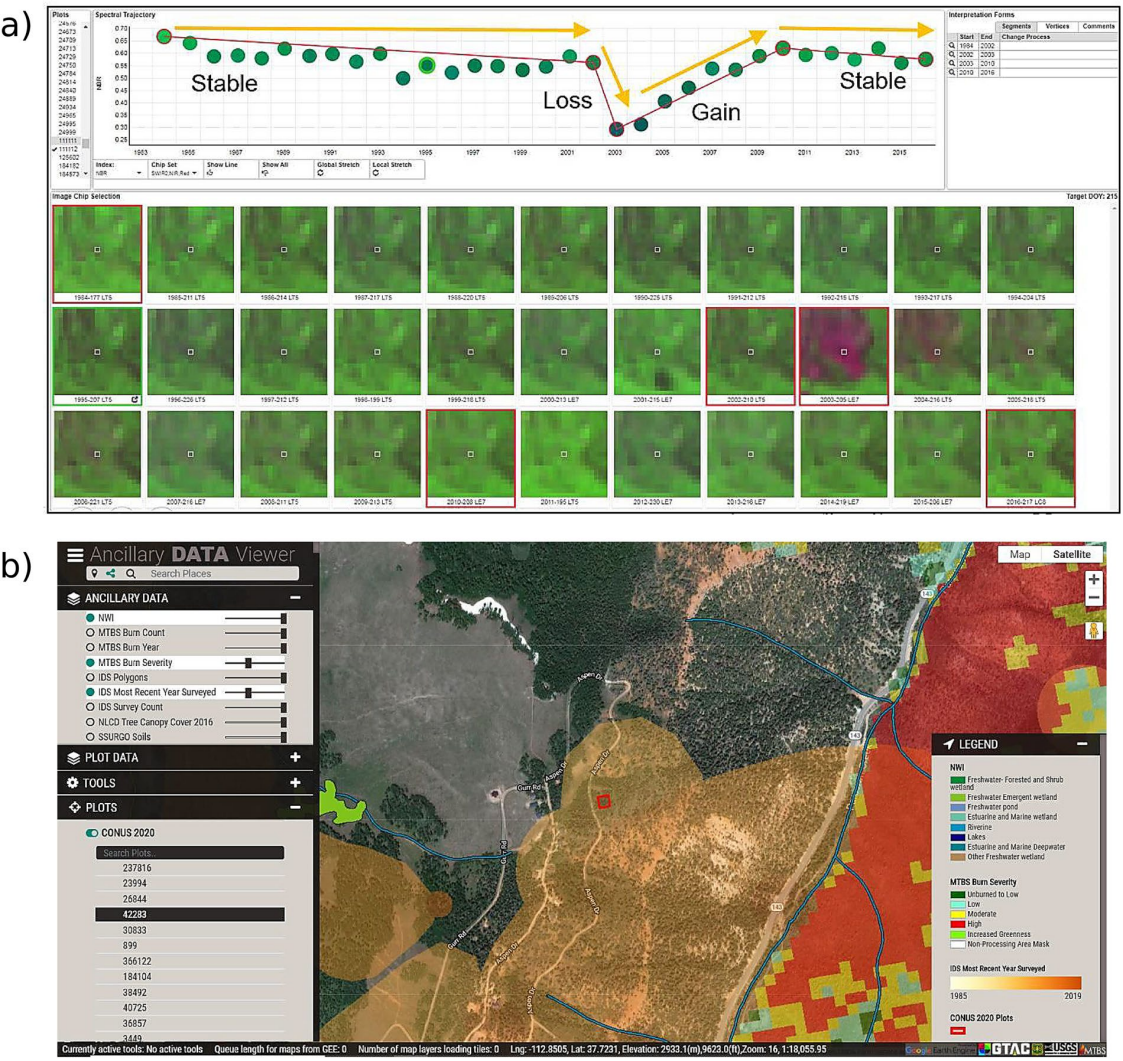
TimeSync Attribution Tool and Ancillary Data Viewer Example. Example of the TimeSync tool (a, top) and the Ancillary Data Viewer (b, bottom). These tools, along with Google Earth Pro, were used in unison to attribute Change Process, Land Cover, and Land Use for each year for each model calibration plot.

Model calibration data were obtained through the TimeSync attribution tool^19^. TimeSync is a web-based platform that enables users to examine time series imagery from Landsat and Sentinel-2, supplemented by high-resolution imagery in Google Earth Pro (https://earth.google.com/) and additional datasets provided though a locally developed Ancillary Data Viewer (http://apps.fs.usda.gov/lcms-viewer/ancillary.html). These resources support the attribution of annual land cover, land use, and change processes at each training point location (Fig. 3).

For LCMS, TimeSync interpretation followed the LCMAP/LCMS Joint Response Design. This framework establishes a standardized approach for assigning a common set of classes related to change process, land cover, and land use (see supplementary materials in Pengra *et al*.^25^). The classes and their definitions are presented below:

- Change Process
  1. Fire: Land altered by fire, regardless of the cause of the ignition (natural or anthropogenic), severity, or land use.
  2. Harvest: Forest land where trees, shrubs, or other vegetation have been severed or removed by anthropogenic means. Examples include clearcutting, salvage logging after fire or insect outbreaks, thinning, and other forest management prescriptions (e.g., shelterwood/seedtree harvest).
  3. Mechanical: Non-forest land where trees, shrubs, or other vegetation has been mechanically severed or removed by chaining, scraping, brush sawing, bulldozing, or any other methods of non-forest vegetation removal.
  4. Structural Decline: Land where trees or other woody vegetation is physically altered by unfavorable growing conditions brought on by non-anthropogenic or non-mechanical factors. This type of loss should generally create a trend in the spectral signal(s) (e.g., Normalized Difference Vegetation Index (NDVI) decreasing, wetness decreasing, shortwave-infrared (SWIR) increasing, etc.). However, the trend can be subtle. Structural decline occurs in woody vegetation environments, most likely from insects, disease, drought, acid rain, etc. Structural decline can include defoliation events that do not result in mortality, such as in spongy moth and spruce budworm infestations, which may recover within one or two years.
  5. Spectral Decline: A plot where the spectral signal shows a trend in one or more of the spectral bands or indices (e.g., NDVI decreasing, wetness decreasing; SWIR increasing; etc.). Examples include cases where: a) non-forest/non-woody vegetation shows a trend suggestive of decline (e.g. NDVI decreasing, wetness decreasing; SWIR increasing; etc.); or b) woody vegetation shows a decline trend that is not related to the loss of woody vegetation, such as when mature tree canopies close resulting in increased shadowing, when species composition changes from conifer to hardwood, or when a dry period (as opposed to stronger, more acute drought) causes an apparent decline in vigor, but no loss of woody material or leaf area.
  6. Wind/Ice: Land (regardless of use) where vegetation is altered by wind from hurricanes, tornados, storms, and other severe weather events, including freezing rain from ice storms.
  7. Hydrology: Land where flooding has significantly altered woody cover or other land cover elements regardless of land use (e.g., new mixtures of gravel and vegetation in and around streambeds after a flood).
  8. Debris: Land (regardless of use) altered by natural material movement associated with landslides, avalanches, volcanos, debris flows, etc.
  9. Other: Land (regardless of use) where the spectral trend or other supporting evidence suggests a disturbance or change event has occurred, but the definitive cause cannot be determined, or the type of change fails to meet any of the change process categories defined above.
  10. Growth/Recovery: Land exhibiting an increase in vegetation cover due to growth and succession over one or more years. Applicable to any areas that may express spectral change associated with vegetation regrowth. In developed areas, growth can result from maturing vegetation and/or newly installed lawns and landscaping. In forests, growth includes vegetation growth from bare ground, as well as the over topping of intermediate and co-dominate trees and/or lower-lying grasses and shrubs. Growth/recovery segments recorded following forest harvest will likely transition through different land cover classes as the forest regenerates. For these changes to be considered growth/recovery, spectral signal should closely adhere to an increasing trend line (e.g., a positive slope that would, if extended to ~20 years, be on the order of .10 units of NDVI) that persists for several years.
  11. Stable: Where no significant change is evident in the spectral signal and the trend is essentially flat. Agricultural systems and wetlands are commonly highly variable spectrally through time and are thus considered stable but ephemeral.
- Land Cover
  1. Tree: Live or standing dead trees.
  2. Tall Shrub (SEAK only): Shrubs greater than 1 m in height (added to the Joint Response design for LCMS TimeSync data collection in Alaska).
  3. Shrub: Shrubs.
  4. Grass/Forb/Herbaceous: Perennial grasses, forbs, or other forms of herbaceous vegetation.
  5. Barren or Impervious: a) Bare soil exposed by disturbance (e.g., soil uncovered by mechanical clearing or forest harvest), as well as perennially barren areas such as deserts, playas, rock outcroppings (including minerals and other geologic materials exposed by surface mining activities), sand dunes, salt flats, and beaches. Roads made of dirt and gravel are also considered barren; or b) man-made materials that water cannot penetrate, such as paved roads, rooftops, and parking lots.
  6. Snow or Ice: Snow and/or ice.
  7. Water: Water.

- Land Use

1. Agriculture: Land used to produce food, fiber, and fuels which is in either a vegetated or non-vegetated state. This includes but is not limited to cultivated and uncultivated croplands, hay lands, orchards, vineyards, confined livestock operations, and areas planted for the production of fruits, nuts, or berries. Roads used primarily for agricultural use (i.e., not used for public transport from town to town) fall in the agriculture land use class.
2. Developed: Land covered by man-made structures (e.g., high density residential, commercial, industrial, mining or transportation), or a mixture of both vegetation (including trees) and structures (e.g., low density residential, lawns, recreational facilities, cemeteries, transportation and utility corridors, etc.), including any land functionally altered by human activity.
3. Forest: Land that is planted or naturally vegetated and that contains (or is likely to contain) 10 percent or greater tree cover at some time during a near-term successional sequence. This may include deciduous, evergreen, and/or mixed categories of natural forest, forest plantations, and woody wetlands.
4. Non-Forest Wetland: Lands adjacent to or within a visible water table (either permanently or seasonally saturated) dominated by shrubs or persistent emergents. These wetlands may be situated shoreward of lakes, river channels, or estuaries; on river floodplains; in isolated catchments; or on slopes. They may also occur as prairie potholes, drainage ditches, and stock ponds in agricultural landscapes and may also appear as islands in the middle of lakes or rivers. Other examples also include marshes, bogs, swamps, quagmires, muskegs, sloughs, fens, and bayous.
5. Other: Lands that are perennially covered with snow and ice, water, salt flats and other undeclared classes. Glaciers and ice sheets or places where snow and ice obscure any other land cover calls are included (assumed is the presence of permanent snow and ice). Water includes rivers, streams, canals, ponds, lakes, reservoirs, bays, or oceans. This assumes permanent water (which can be in some state of flux due to ephemeral changes brought on by climate or anthropogenic).
6. Rangeland or Pasture: This class includes any area that is either: a) rangeland, where vegetation is a mix of native grasses, shrubs, forbs and grass-like plants largely arising from natural factors and processes such as rainfall, temperature, elevation, and fire, although limited management may include prescribed burning as well as grazing by domestic and wild herbivores; or b) pasture, where vegetation may range from mixed, largely natural grasses, forbs, and herbs to more managed vegetation dominated by grass species that have been seeded and managed to maintain near monoculture.

Many of these classes have definitions that are difficult to consistently interpret using 30-m spatial resolution satellite data, Google Earth Pro, and other ancillary data that are inconsistently available in space and time. Additionally, manually attributing plots with the correct change process, land cover, and land use over many years is tedious and error-prone^23^. While most plots are straight-forward and do not experience change, some experience a variety of change processes and/or mixed covers and uses that present challenges to definitively attribute. These plots are flagged for further inspection by analysts and reviewed manually to reach a final attribution. We acknowledge this limitation to TimeSync and the Joint Response Design. Despite this limitation, presently, this method serves as the best available method for collecting long-term time-series reference data^19,23,30,31^. LCMS is actively pursuing improvements to the implementation of this response design using TimeSync and accompanying quality assurance methods.

#### Different thematic levels to address different needs

LCMS employs several cross-walking methods to combine the classes measured with TimeSync (above) in different ways to meet the need for varying levels of thematic detail and to address limitations to consistent distinctions between similar classes (notably Structural and Spectral decline). In general, products with higher numbers of classes exhibit lower levels of accuracy^32^. Users should use the level that best matches their error tolerance and need for thematic detail.

The delivered map classes are the highest level (first column) in Tables 2–4. Table 2 shows the change processes cross-walked into four final change classes (as Level 3): (1) Fast Loss; (2) Slow Loss; (3) Gain; and (4) Stable. In LCMS pilot projects, modeling all Change Process classes failed to produce acceptable results. We therefore bin TimeSync-attributed Change Processes to Fast Loss, Slow Loss, and Gain at the highest level to allow for a usable product. The respective definitions of each Change class for each Level can be derived from the Change Process definitions above and Table 2.

**Table 2:** Change Process classes cross-walked into final classes (Level 3). Lower Change Levels 1 and 2 are provided as a standard method to further bin classes.

TimeSync Change Process	Final Class (Level 3)	Final Class (Level 2)	Final Class (Level 1)
**Structural decline**	Slow Loss	Loss	Loss
**Spectral decline**	Slow Loss	Loss	Loss
**Fire**	Fast Loss	Loss	Loss
**Harvest**	Fast Loss	Loss	Loss
**Mechanical**	Fast Loss	Loss	Loss
**Wind/Ice**	Fast Loss	Loss	Loss
**Hydrology**	Fast Loss	Loss	Loss
**Debris**	Fast Loss	Loss	Loss
**Other**	Fast Loss	Loss	Loss
**Growth/Recovery**	Gain	Gain	Stable
**Stable**	Stable	Stable	Stable

**Table 3:** List of primary and secondary Land Cover classes modeled in LCMS. Successional classes are grouped and highlighted with italic font.

TimeSync Land Cover Primary	TimeSync Land Cover Secondary	Final Class (Level 4)	Final Class (Level 3)	Final Class (Level 2)	Final Class (Level 1)
**Tree**	NA	Tree	Tree	Tree Vegetated	Vegetated
**Tall Shrub**	*Tree*	Tall Shrub and Tree Mix	Tree	Tree Vegetated	Vegetated
**Shrub**	*Tree*	Shrub and Tree Mix	Tree	Tree Vegetated	Vegetated
**Grass/Forb/Herb**	*Tree*	Grass/Forb/Herb and Tree Mix	Tree	Tree Vegetated	Vegetated
**Barren**	*Tree*	Barren and Tree Mix	Tree	Tree Vegetated	Vegetated
**Tall Shrub**	NA	Tall Shrub	Shrub	Non-Tree Vegetated	Vegetated
**Shrub**	NA	Shrub	Shrub	Non-Tree Vegetated	Vegetated
**Grass/Forb/Herb**	*Shrub*	Grass/Forb/Herb and Shrub Mix	Shrub	Non-Tree Vegetated	Vegetated
**Barren**	*Shrub*	Barren and Shrub Mix	Shrub	Non-Tree Vegetated	Vegetated
**Grass/Forb/Herb**	NA	Grass/Forb/Herb	Grass/Forb/Herb	Non-Tree Vegetated	Vegetated
**Barren**	*Grass/Forb/Herb*	Barren and Grass/Forb/Herb Mix	Grass/Forb/Herb	Non-Tree Vegetated	Vegetated
**Barren or Impervious**	NA	Barren or Impervious	Barren or Impervious	Non-Vegetated	Non-Vegetated
**Snow or Ice**	NA	Snow or Ice	Snow or Ice	Non-Vegetated	Non-Vegetated
**Water**	NA	Water	Water	Non-Vegetated	Non-Vegetated

The Snow or Ice and Water classes are not modeled with any secondary land cover classes since they are not likely to be part of vegetation succession. Lower Land Cover Levels are provided as a standard method to further bin classes.

**Table 4:** List of Land Use classes modeled in LCMS (represented as Level 3 without applying a cross-walk).

TimeSync Land Use	Final Class (Level 3)	Final Class (Level 2)	Final Class (Level 1)
**Agriculture**	Agriculture	Agriculture	Anthropogenic
**Developed**	Developed	Developed	Anthropogenic
**Forest**	Forest	Forest	Non-Anthropogenic
**Non-Forest Wetland**	Non-Forest Wetland	Other	Non-Anthropogenic
**Other**	Other	Other	Non-Anthropogenic
**Rangeland or Pasture**	Rangeland or Pasture	Rangeland or Pasture	Non-Anthropogenic

Lower Land Use Levels 1 and 2 are provided as a standard method to further bin classes.

Land Cover requires a distinct cross-walking methodology. Each TimeSync plot is assigned a primary land cover class representing the majority of the plot. During data collection, any additional land cover class occupying 10 percent or more of the plot is designated as a secondary land cover class. Because a plot may contain between zero and five secondary land cover classes, only primary/secondary pairings in which the secondary class represents a higher position along the land cover successional trajectory are cross-walked. The expected successional sequence, from lowest to highest is: Barren to Grass/Forb/Herb, Grass/Forb/Herb to Shrub, and Shrub to Tree.

Within LCMS, primary/secondary Land Cover combinations are cross-walked to a single class, represented in Table 3 as Level 4. Lower Land Cover Levels (1–3) are used to progressively aggregate classes into broader categories to reduce mapping error, but they are not explicitly applied in model development.

For Land Use modeling, we use the Land Use classes—Agriculture, Developed, Forest, Non-Forest Wetland (NFW), Other, and Rangeland or Pasture—directly from TimeSync without cross-walking (as Level 3); lower Land Use Levels 1-2 are also provided as a standard method to balance thematic detail and accuracy (Table 4). Level 1 classes attempt to characterize uses in the broadest level we use our lands. It is true that there are instances of anthropogenic use in Forest (plantations), Rangeland or Pasture (pasture is actively managed), and even Other (reservoirs). However, we want to provide a product level to present users more intense anthropogenic land uses relative to those that are likely less influenced by humans.

### Model predictor data

We incorporate spectral inputs from Landsat and Sentinel-2 imagery along with topographic data from the United States Geological Survey (USGS) 3D Elevation Program (3DEP)^33^ for modeling. The following sections provide detailed descriptions of each dataset.

#### Remote sensing spectral data

LCMS relies on USGS Collection 2 Tier 1 Landsat 4, 5, 7, 8, and 9, together with Sentinel-2a and -2b level 1 C top-of-atmosphere reflectance data. When combining Landsat and Sentinel-2 inputs, surface reflectance products are not used—the Sentinel-2 surface reflectance data available within GEE are terrain-corrected, while Landsat surface reflectance data are not terrain-corrected.

For cloud and cloud-shadow masking of Landsat imagery, we applied the CFmask algorithm (an implementation of Fmask^34^), the cloudScore algorithm^35^, and the Temporal Dark Outlier Mask (TDOM) method^36^. For Sentinel-2 imagery spanning 2016–2022, masking was performed using the cloud probability product (S2 Cloudless^37^) in conjunction with TDOM. Beginning in 2023, cloud and cloud-shadow masking for Sentinel-2 data transitioned to the Cloud Score + algorithm^38^. All procedures for remote sensing data preparation are available within the GTAC GEE data processing and visualization library geeViz (https://geeviz.org).

LCMS uses cloud- and cloud shadow-masked observations together with annual composites to support time series processing. Annual composites are the geometric medoid of all unmasked observations within a specified date range for each year^10^. Because data availability and seasonal patterns vary across regions and time, the compositing window is adjusted accordingly (Table 5). In CONUS, the recent availability of Sentinel-2 imagery enabled a shorter compositing period.

**Table 5:** Dates used for annual compositing of Landsat and Sentinel-2 data.

Study Area	Landsat-only (1984–2016) Start Date	Landsat-only (1984–2016) End Date	Landsat and Sentinel-2 (2017-present) Start Date	Landsat and Sentinel-2 (2017-present) End Date
**CONUS**	June 1	September 30	July 1	September 1
**SEAK**	June 15	September 15	June 15	September 15
**PRUSVI**	June 1	May 31	June 1	May 31
**HI**	January 1	December 31	January 1	December 31

The geometric medoid represents the observation that minimizes the sum of the square difference (Euclidean distance) from the median value across all bands—selecting the most central value in the multi-dimensional feature space. For each pixel, all band values used for the medoid are drawn from same acquisition date. The feature space includes the green, red, near-infrared (NIR), first shortwave-infrared (SWIR1), and second shortwave-infrared (SWIR2) bands. Pixels lacking a cloud-free or cloud shadow-free observation for a given year are null for that year’s composite. The bands, indices, and transformations incorporated in subsequent steps are: (1) blue; (2) green; (3) red; (4) NIR; (5) SWIR1; (6) SWIR2; (7); Normalized Difference Vegetation Index (NDVI)^39^; (8) Normalized Burn Ratio (NBR)^40^; (9) Normalized Difference Moisture Index (NDMI)^41^; (10) Normalized Difference Snow Index (NDSI); Tasseled Cap brightness, greenness, wetness^42^ and brightness/greenness angle^30^.

#### Time series processing

The objective of time series processing is to identify intervals that share similar land cover, land use and/or change processes. Because individual methods have distinct strengths and limitations, LCMS applies the ensemble approach described in Cohen *et al*.^43^ and Healey *et al*.^13^. In its current operational form, LCMS uses LandTrendr^9,10^ and CCDC^11^ to segment the processed time series. Most simply, LandTrendr identifies changes in linear trends, while CCDC identifies changes in phenology (seasonality). By incorporating two fundamentally different change detection approaches, LCMS models gain robust predictors for Change, Land Cover, and Land Use. LandTrendr generally operates with a single annual observation, while CCDC leverages all available cloud- and cloud shadow-free observation from the time series^44^.

#### LandTrendr methods

LandTrendr recursively breaks an annual time series into a set of segments^9^. This reduces noise in the time series and provides attributes of each segment—such as the duration and slope. We use the following information from LandTrendr for each spectral band/index for each year:

- Fitted value
- Difference of that year’s fitted value from the fitted value of the start vertex
- Difference from the start to end fitted value of the segment that year falls in
- The duration of the segment that year falls in
- The slope of the segment that year falls in

LCMS uses the GEE version of LandTrendr and parameters outlined in Kennedy *et al*.^10^ (Table 6 – adapted from the GEE LandTrendr documentation). Starting in v2023.9, we increased the maxSegments parameter from 6 to 9. We made this change after noticing some appropriate breaks were often missed by LandTrendr in the pilot v2023.9 LandTrendr run. Increasing the maxSegments to 9 addressed this issue.

**Table 6:** LandTrendr parameters used.

Parameter Name	Value	Description
**maxSegments**	9	Maximum number of segments to be fitted on the time series.
**spikeThreshold**	0.9	Threshold for damping the spikes (1.0 means no damping).
**vertexCountOvershoot**	3	The initial model can overshoot the maxSegments + 1 vertices by this amount. Later, it will be pruned down to maxSegments + 1.
**preventOneYearRecovery**	False	Prevent segments that represent one-year recoveries.
**recoveryThreshold**	0.25	If a segment has a recovery rate faster than 1/recoveryThreshold (in years), then the segment is disallowed.
**pvalThreshold**	0.05	If the p-value of the fitted model exceeds this threshold, then the current model is discarded and another one is fitted using the Levenberg-Marquardt optimizer.
**bestModelProportion**	0.75	Takes the model with most vertices that has a p-value that is at most this proportion away from the model with lowest p-value.

Further documentation of the LandTrendr method used can be found in the GEE reference documentation^45^.

#### CCDC methods

CCDC partitions the time series by detecting outliers relative to a harmonic regression model. Harmonic regression is useful for highlighting distinct seasonality signatures of different land cover and/or land use types. A significant departure from the seasonality signature signals a change. A new harmonic model is then fit after the period of change^11^.

Input data include all Landsat observations free of cloud and cloud shadows, identified using the CFmask algorithm^34^. For CONUS, we employ surface reflectance data for CCDC. It was later discovered that the surface reflectance correction algorithm performs poorly over snow, ice, and water^46^, yielding reflectance values that are frequently outside the valid range of 0-1. Consequently, we use top-of-atmosphere reflectance data in other study areas in CCDC. Sentinel-2 data are excluded due to memory constraints within GEE when processing the full archive of Landsat and Sentinel-2 imagery from 1984 onward.

The GEE version of CCDC^47^ is used for LCMS. The parameters used are shown in Table 7 (adapted from the GEE CCDC documentation). We use the same bands to find the medoid value for annual composites as *breakpointBands*. The fractional years as the *dateFormat* is easier to use and apply to the harmonic model. *Lambda* is the default value divided by 10000 to match the scaling of the reflectance values. *MaxIterations* had to be reduced to 10000 to help reduce memory errors within GEE.

**Table 7:** Continuous Change Detection and Classification (CCDC) parameters used.

Parameter Name	Value	Description
**breakpointBands**	[“green”, “red”, “NIR”, “SWIR1”, “SWIR2”]	The name or index of the bands to use for change detection. If unspecified, all bands are used.
**tmaskBands**	null	The name or index of the bands to use for iterative TMask cloud detection. These are typically the green band and the SWIR2 band. If unspecified, TMask is not used. If specified, ‘tmaskBands’ must be included in ‘breakpointBands’.
**minObservations**	6	The number of observations required to flag a change.
**chiSquareProbability**	0.99	The chi-square probability threshold for change detection in the range of [0, 1]
**minNumOfYearsScaler**	1.33	Factors of minimum number of years to apply new fitting.
**dateFormat**	1	The time representation to use during fitting: 0 = jDays, 1 = fractional years, 2 = unix time in milliseconds. The start, end and break times for each temporal segment will be encoded this way.
**lambda**	0.002	Lambda for LASSO regression fitting. If set to 0, regular OLS is used instead of LASSO.
**maxIterations**	10000	Maximum number of runs for LASSO regression convergence. If set to 0, regular OLS is used instead of LASSO.

LCMS uses all cosine and sine coefficients from the first three harmonics (2π – 1 cycle/year, 4π – 2 cycles/year, and 6π – 3 cycles/year) from the CCDC outputs. By using the first 3 harmonics, the model can fit a variety of phenological patterns—such as a typical single green-up and green-down within a year, as well as cycles with higher frequencies common in some agriculture and grasslands. Instead of using the slope and intercept generated by CCDC, we use the predicted value based on the harmonic model in place of the intercept, and we use the difference between that year and the previous year’s fitted values as the slope.

For each pixel, we use the medoid composite pixel date for each year as the date to predict the CCDC harmonic model. This helps to ensure close alignment between LandTrendr and CCDC values that are used in our models. For any pixel without a valid medoid composite value for a given year, we use the median date for available composite dates for that pixel.

After CCDC finds a break, it does not start a new harmonic model until a period of relative stability is observed^11^. This introduces gaps between segments. For these gaps, we use the next segment after the break date during the gap period. Often, breaks near the end of the time series have not resumed a period of relative stability and therefore lack the next segment needed to fill the gap. In this scenario, we allow the previous segment to extend by as much as 0.3 of a year. If there is still no segment available prior to 0.3 year before the pixel’s composite date, that pixel is excluded from the map for that year. This allows CCDC to work properly within the LCMS annual ensemble framework.

CCDC initializes its model with statistics from the entire time series for each pixel^11^. Therefore, if a single additional year is added, it is quite likely all segments will be slightly different, despite only adding a single year at the end. Additionally, at present, there is no way to simply extend a current CCDC output with additional observations. To avoid any sudden jumps in CCDC values, the CCDC algorithm must be re-run annually to be consistent with the LCMS period from 1984 to the present year—a computationally expensive process.

**Fig. 4:**
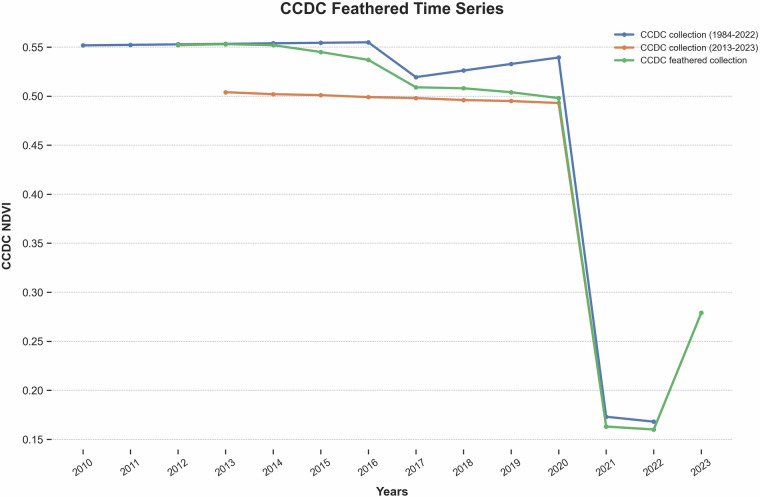
CCDC Feathering Example. An example time series showing the feathering of two Continuous Change Detection and Classification (CCDC) image collections for a single pixel. The blue line is Normalized Difference Vegetation Index (NDVI) from the v2022.8 CCDC collection that spans 1984–2022, and the orange line is NDVI from the v2023.9 CCDC collection that spans 2013–2023. The green line is the weighted average of the two CCDC collections used in our models.

From 2020–2022, we reran CCDC annually for all study areas. This proved expensive and computationally inefficient. To streamline CCDC re-processing, we developed a method that reuses prior CCDC outputs and integrates them with a newer, shorter-duration run of CCDC. For CONUS and SEAK v2023.9, we ran CCDC algorithm for 2013–2023 and feathered (blended) it with the existing CCDC dataset from 1984–2022 (the v2022.8 CCDC collection). This feathered approach combines the original CCDC collection data from 1984–2012 with a linearly weighted average of both the previous and updated CCDC outputs from 2013–2021 (Fig. 4). The weighting gradually shifts from the older to the newer collection, starting at 0 in 2013 and reaching 1 by 2021, with 2022 fully sourced from the updated run. This technique helps reduce inconsistencies or artifacts that can arise from overlapping CCDC outputs. For HI and PRUSVI, given their smaller spatial extent, we opted to generate new 1984–2023 CCDC runs.

Further documentation of the CCDC methods used can be found in the GEE reference documentation^47^.

#### Terrain data

LCMS incorporates terrain metrics to provide elevation, slope, aspect, and slope-position information to the model. The variables included are:

- Elevation
- Sine (Aspect)
- Cosine (Aspect)
- Slope
- Slope-position (calculated using circular kernels with 11-, 21-, and 41-pixel window)^48^

Across all study areas, terrain data were derived from the 10 m USGS 3DEP dataset^34^, with resampling performed using cubic convolution.

#### Summary

All variables described in this section are incorporated into the methods outlined below. A complete list of predictor variables considered for modeling is provided in Table 8.

**Table 8:** List of Landscape Change Monitoring System (LCMS) model predictor variables.

LANDTRENDR (Landsat and Sentinel 2) (Annual Values)								
	Fitted	Difference	Duration	Magnitude	Slope			
**Spectral Bands**								
**blue**	✓	✓	✓	✓	✓			
**green**	✓	✓	✓	✓	✓			
**red**	✓	✓	✓	✓	✓			
**NIR**	✓	✓	✓	✓	✓			
**SWIR1**	✓	✓	✓	✓	✓			
**SWIR2**	✓	✓	✓	✓	✓			
**Indices**								
**NDVI**	✓	✓	✓	✓	✓			
**NBR**	✓	✓	✓	✓	✓			
**NDMI**	✓	✓	✓	✓	✓			
**NDSI**	✓	✓	✓	✓	✓			
**Tasseled Cap Transformation**								
**brightness**	✓	✓	✓	✓	✓			
**greenness**	✓	✓	✓	✓	✓			
**wetness**	✓	✓	✓	✓	✓			
**brightness/greenness angle**	✓	✓	✓	✓	✓			
**CCDC (Landsat Only) (Annual Values)**								
	**Fitted**	**Diff**	**COS 1**	**COS 2**	**COS 3**	**SIN 1**	**SIN 2**	**SIN 3**
**Spectral Bands**								
blue	✓	✓	✓	✓	✓	✓	✓	✓
green	✓	✓	✓	✓	✓	✓	✓	✓
red	✓	✓	✓	✓	✓	✓	✓	✓
NIR	✓	✓	✓	✓	✓	✓	✓	✓
SWIR1	✓	✓	✓	✓	✓	✓	✓	✓
SWIR2	✓	✓	✓	✓	✓	✓	✓	✓
**Indices**								
NDVI	✓	✓	✓	✓	✓	✓	✓	✓
**Terrain (Single Value)**								
Elevation	✓							
Slope	✓							
cos(Aspect)	✓							
sin(Aspect)	✓							
TPI (11 pixel)	✓							
TPI (21 pixel)	✓							
TPI (41 pixel)	✓							

Annual values are different for each year of the analysis period, while the single-value terrain variables remain constant.

### Modeling (Supervised Classifications)

We use the random forest modeling method^21^ for all supervised classifications in LCMS. For raster-based classification, LCMS employs the GEE instance of random forests, “smileRandomForest”^49^, and we use sklearn.ensemble.RandomForestClassifier^50^ for predictor variable selection and map validation.

Separate random forest models are developed for each LCMS product—Change, Land Cover, and Land Use—for each study area. While many CONUS-wide mapping programs employ multiple regionalized models^1,51,52^ for CONUS, LCMS cannot implement such an approach due to scarcity of model calibration and validation data. The raw annual model output is the proportion of trees within the random forest model that was chosen for each class. For example, if the Change random forest model consists of 100 classification trees and, in 2005, 35 of those trees select Fast Loss, 10 select Slow Loss, 5 select Gain, and 50 select Stable, that pixel receives a value of 0.35 for Fast Loss, 0.10 for Slow Loss, 0.05 for Gain, and 0.5 for Stable in 2005. These decision tree agreement scores are referred to as “model confidence,” acknowledging that unanimity among trees may reflect biases in our TimeSync-based reference data and does not constitute a formal measure of uncertainty. Model confidence ranges from 0 and 1 and is available for each product annually from 1985 through the most recent completed growing season.

#### Predictor variable selection

To minimize predictor variable co-variation and exclude variables that do not enhance the model, we apply a two-step filtering process. First, predictor pairs with a Pearson’s correlation coefficient (r) exceeding an absolute value greater than 0.95 are removed (pandas.DataFrame.corr^53^). Any variable with a mean absolute r greater than 0.95 across all pairs is dropped. Second, recursive feature elimination is conducted using a five-fold grouped cross-validation (sklearn.feature_selection.RFECV^54^). The variable set yielding the highest accuracy is retained for each model.

#### Hyperparameter tuning and change thresholds

We apply a 10-fold grid search grouped cross-validation (sklearn.model_selection.GridSearchCV^55^) to identify the optimal combination of random forest hyperparameters to maximize cross-validation accuracy for each LCMS model (Change, Land Cover, and Land Use). These parameters include number of trees, minimum samples per leaf, and maximum number of features.

For the Change model, optimum model confidence thresholds for each class were determined by evaluating precision and recall at all possible thresholds (from 0–100) and selecting the threshold that maximizes both metrics, as measured by the F1 score.

### Map assembly

**Fig. 5:**
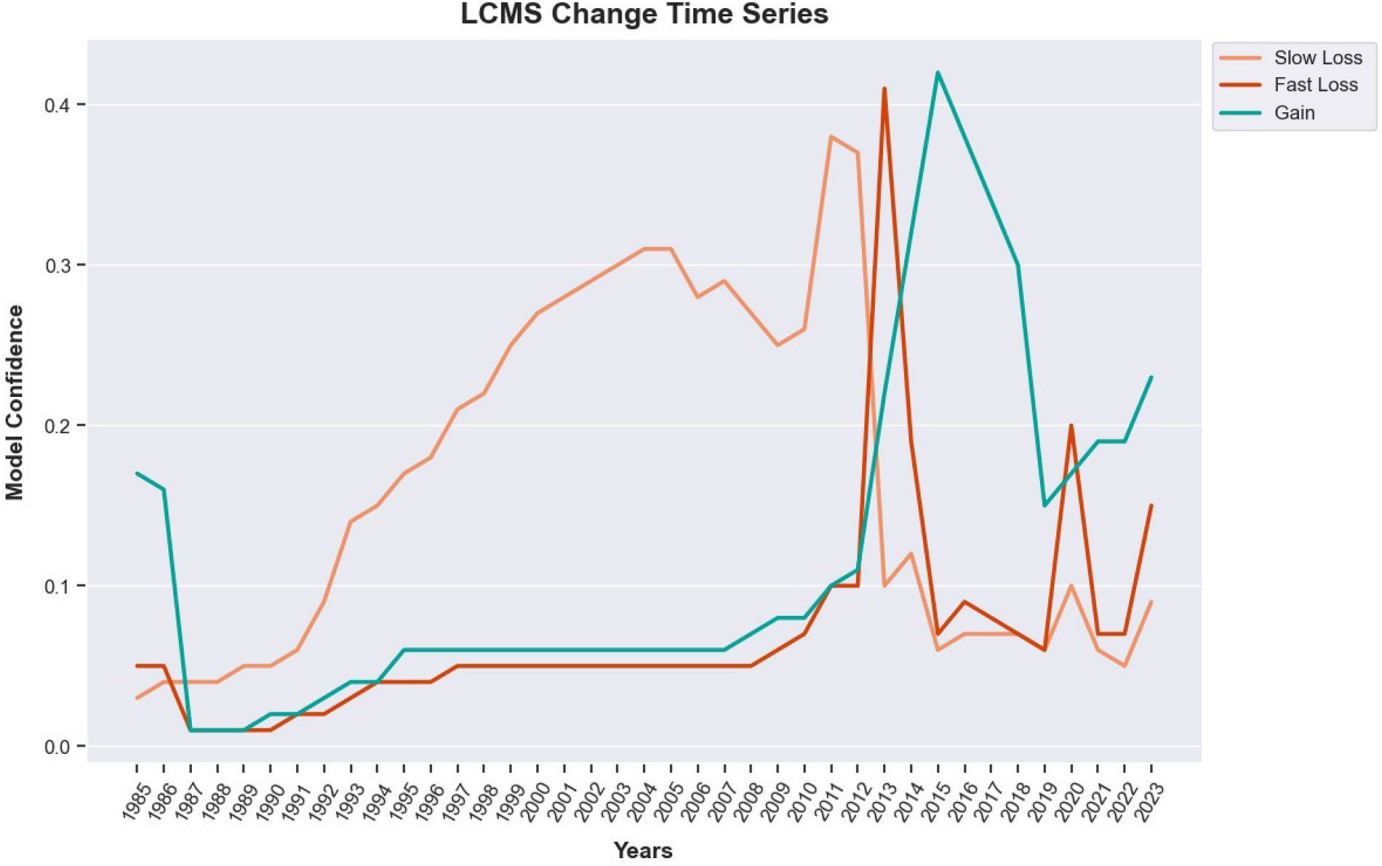
Raw Change Model Confidence Values. Landscape Change Monitoring System Change model confidence values for a single pixel.

As described above, each class within the Change, Land Cover, and Land Use products is assigned a model confidence score, defined as the proportion of trees within the random forest model that classify a given pixel as that class. Figure 5 illustrates an example time series of Change model confidence for a single pixel. For each year, the class with the highest confidence is initially selected for the LCMS product (Change, Land Cover, and Land Use). Because the Stable class is not explicitly modeled, the highest confidence line must also exceed the threshold established for that class.

To minimize commission and omission errors, we apply a series of probability thresholds and rulesets informed by ancillary datasets. These datasets include the Global Human Settlement (GHSL)^28^ built-up surface for all study areas, the U.S. Department of Agriculture National Agricultural Statistics Service Cropland Data Layer (CDL)^56,57^ for CONUS, and the Global Surface Water Mapping Layer (JRC Water)^58^ for HI. Map assembly rules were developed primarily for Land Use, with additional Land Cover rules applied in SEAK (to restrict Tree and Snow Land Cover classes in the extensive intertidal zones at sea level), and in HI (to limit Grass and Barren commission in reef areas in the ocean). A single Change rule was applied across all study areas to prevent Change in non-vegetated land cover classes. The complete set of rules and associated ancillary datasets is summarized in Supplementary Table 1. Final pixel classification is determined by the highest probability class that meets the minimum threshold defined by these rules.

We acknowledge these assembly rules make it difficult to directly relate raw model confidence to final mapped classes. Therefore, in addition to the assembled output we also provide the raw model output to enable users with specific needs to introduce their own assembly rules. For example, if a user needs a Fast Loss layer with low omission error, they could take the Fast Loss confidence and apply a very low threshold. Conversely, if false positives were costly, a user could apply a high threshold.

## Data Records

LCMS data are distributed in three different locations to meet different user needs^59–61^ (Table 9).

**Table 9:** LCMS data distribution locations, available data products, and format.

Name	URL	Annual	Change Summary	Raw Model Confidence	Format	Additional Information
Forest Service FSGeodata Clearinghouse	https://data.fs.usda.gov/geodata/rastergateway/LCMS	X	X		Cloud Optimized GeoTIFF (COG)	Annual COG for each study area, for each product and Change summary products for each study area. Each output is delivered as a zip file. It contains the raster data as a Cloud-Optimized GeoTIFF, XML ESRI ISO 19139 metadata, HTML ISO 19139 metadata, and Class name XML.
Google Earth Engine Data Catalog	https://developers.google.com/earth-engine/datasets/catalog/USFS_GTAC_LCMS_v2023-9	X		X	Earth Engine ImageCollection	GEE ImageCollection of annual images for each study area. Each image contains the three products as well as raw model confidence for each class and QA bits as individual bands.
ESRI Image Services	https://apps.fs.usda.gov/fsgisx01/rest/services/RDW_LandscapeAndWildlife	X	X		REST Image Service	Annual time-enabled REST image service for each study area, for each product and Change summary products.

Notice each location provides a unique data format and/or data output.

LCMS releases new versions annually—CONUS and SEAK are generally published by June and PRUSVI and HI by October. We package the final LCMS deliverables in two ways: annual and summarized layers. For each product (Change, Land Cover, and Land Use) we assemble annual maps at the highest classification level, as discussed above. Lower levels can be cross-walked from the highest level using Tables 2–4. An example of how to do this using Google Earth Engine is provided in the geeViz package (https://geeviz.org/notebooks/LCMS_Levels_Viewer_Notebook). Additionally, we provide summary products for each Change Level 3 class.

To summarize the Change layers, we use two methods: *most recent* and *most probable*. The *most recent* method chooses the year of the respective Change class that occurred most recently, while the *most probable* method chooses the year of the respective Change class with the highest model confidence. The former can be useful for applications that need to know the most recent year a given Change class was present, while the latter is useful for applications that need to know when a given Change event peaked. For example, the time series of Change model confidences, or probabilities, for a given pixel is shown in Fig. 5.

The *most recent* Change years for this example are:

- Slow Loss: 2012
- Fast Loss: 2013
- Gain: 2023
  The *most probable* Change years are:
- Slow Loss: 2011
- Fast Loss: 2013
- Gain: 2015

Generally, the two summary methods differ most for long-term change processes, such as Gain and Slow Loss.

Ancillary information on the origin of the annual LCMS product output values is provided as part of a QA bit layer. This layer includes whether an interpolated value was used to produce the LCMS output, the sensor, and the day of year the value came from. The QA bits are as follows:

- 1: Interpolated (0), not interpolated (1)
- 2–6: Which sensor the pixel came from
  - 4 = Landsat 4
  - 5 = Landsat 5
  - 7 = Landsat 7
  - 8 = Landsat 8
  - 9 = Landsat 9
  - 21 = Sentinel 2a
  - 22 = Sentinel 2b
- 7–15: Which Julian day the pixel came from (1–365)

Bitwise operations can be leveraged to unpack the QA decimal numbers to valid pixel values for the non-interpolated data, sensor, and Julian day (see metadata for a more detailed method).

## Data Overview

### Example outputs

**Fig. 6:**
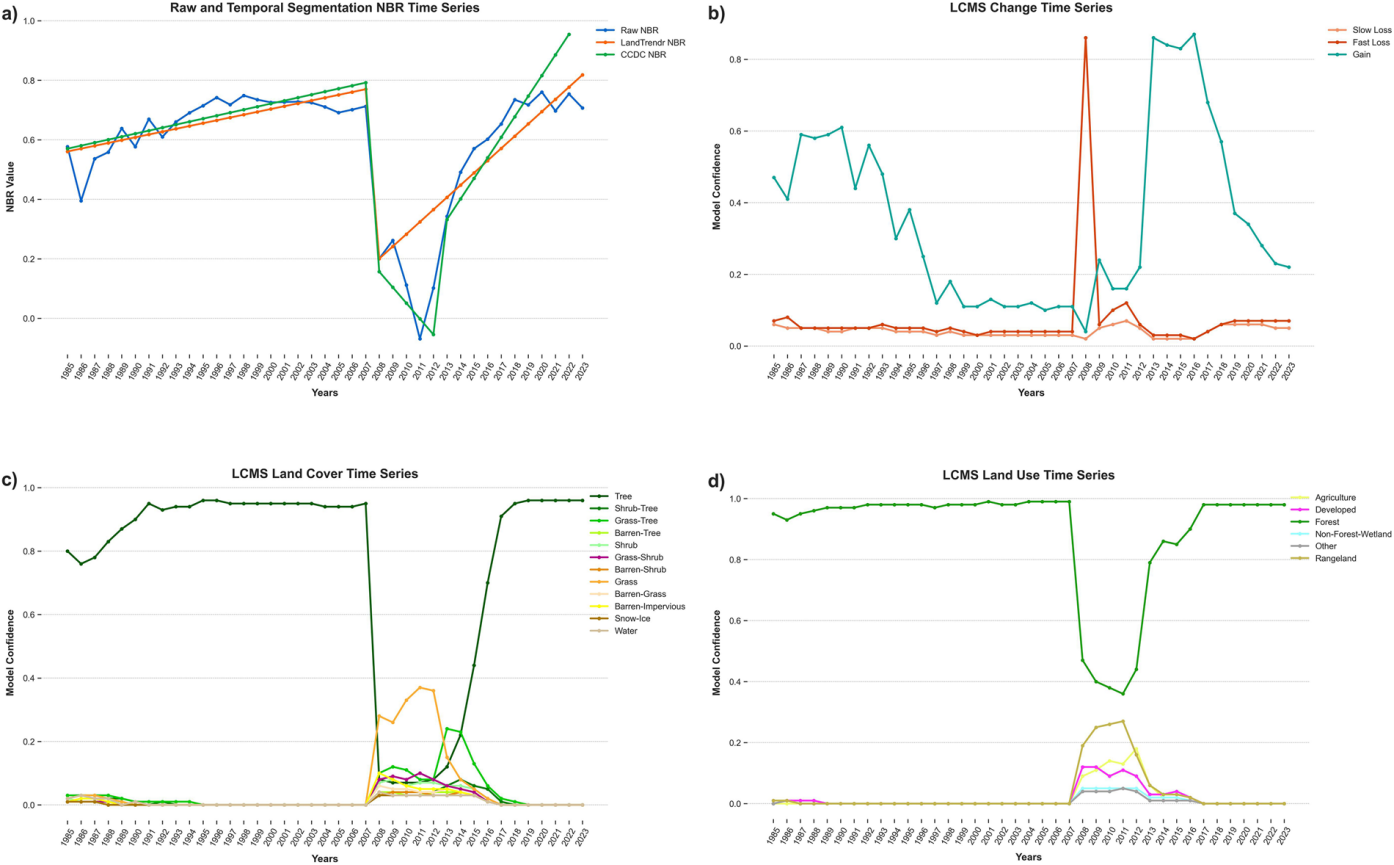
LCMS Raw Model Probability Product Suite Example. An example time series of Landscape Change Monitoring System input predictor values, as well as raw modeled probabilities for each year for a pixel where a fire occurred in 2008. In the upper left chart (**a**), notice how the normalized burn ratio (NBR) remains low for several years after the large decrease—then it rapidly rises. This is reflected in the Change probability time series in the upper right (**b**) and Land Cover in the lower left (**c**). Note that Land Use never changes from Forest, but the model becomes less confident of its use (**d**).

We will present four example output areas to introduce the LCMS dataset. First, we will look at the impact of a fire event on a small subset of the raw predictor data and the resulting raw modeled confidence of Change, Land Cover, and Land Use (Fig. 6). Figure 6a shows one of the vegetation indices—the normalized burn ratio (NBR). NBR is sensitive to changes in moisture. Note how the raw NBR relates to the smoother LandTrendr and CCDC fitted NBR values. There is a large drop in NBR, followed by a prolonged period of low NBR values, followed by a quick increase. This is reflected in the Change (Fig. 6b), Land Cover (Fig. 6c), and Land Use (Fig. 6d) model probabilities. The Change time series (Fig. 6b) shows that the Fast Loss model confidence peaks in the year of the fire (2008) to a value that exceeds the Fast Loss threshold of 0.29. As one might expect with growth and recovery following a severe fire, several years passed before the Gain model confidence rose to levels above the Gain threshold of 0.29 in 2012. Complementing the Change time series, the Land Cover time series (Fig. 6c) shows that the Tree class had a very high model confidence for each year until the fire in 2008. Following the fire, the Shrub model confidence increases. In the following years, we see the probability of Grass/Forb/Herb & Tree mix increase, most likely indicating that there are live trees in this pixel with grasses becoming increasingly prevalent. Tree eventually becomes the most confident class again in 2015. Since a fire generally does not indicate a Land Use transition, the Land Use model’s Forest confidence dips (Fig. 6d) but remains the highest. This is a result of our model calibration data containing many examples of Forest remaining Forest after change events that result in a loss of trees.

This example illustrates the ability of LCMS to answer questions related to land use separately from land cover. Not all changes in land cover result in changes in land use and vice versa. A forest land use can experience a fire and transition land cover types without transitioning land use. Meanwhile, a treed area can be converted from a forest to a developed land use in the form of a park or housing development but remain dominant tree land cover.

**Fig. 7:**
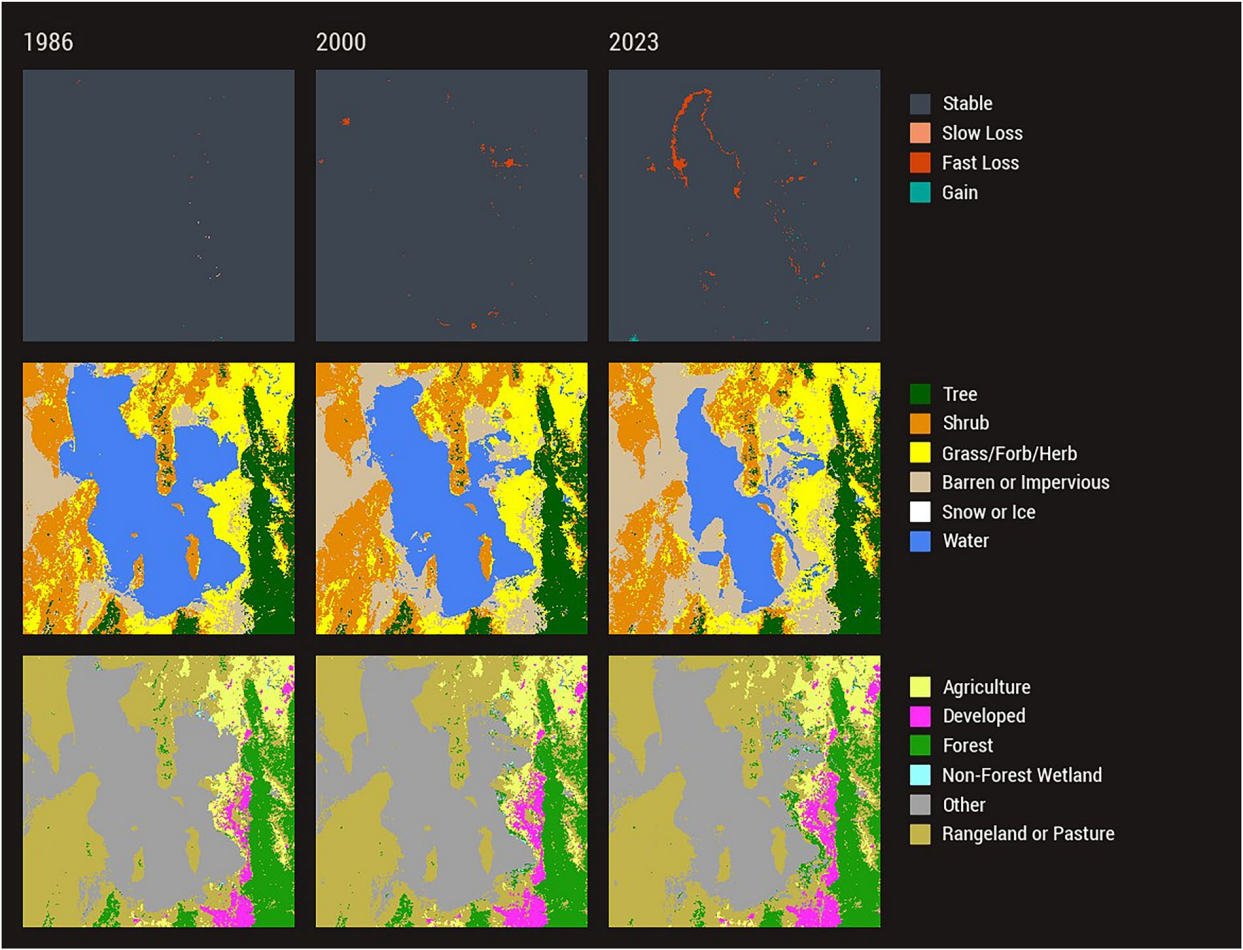
LCMS Product Suite Example – Great Salt Lake. Landscape Change Monitoring System (LCMS) Level 3 Change, Land Cover, and Land Use products illustrating the change in water cover of the Great Salt Lake, Utah. Notice the Land Cover over the Great Salt Lake changes while the Land Use remains largely unchanged.

**Fig. 8:**
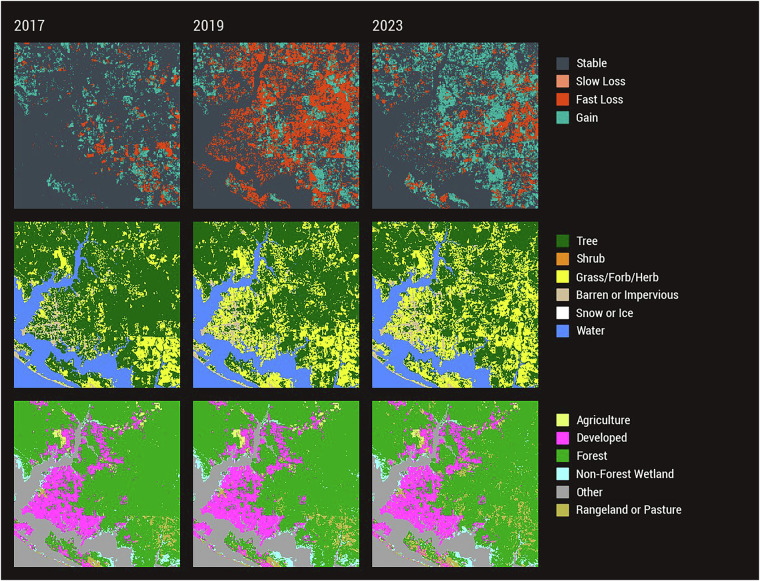
LCMS Product Suite Example – Hurricane Michael. Landscape Change Monitoring System (LCMS) Level 3 Change, Land Cover, and Land Use products illustrating the effects of Hurricane Michael (October 2018) near Pensacola, Florida. Widespread Fast Loss is seen in the year following the Category 5 storm with subsequent years seeing vegetation cover Gain. Land Cover transitions from Tree to Grass/Forb/Herb, while Land Use remains largely unchanged.

**Fig. 9:**
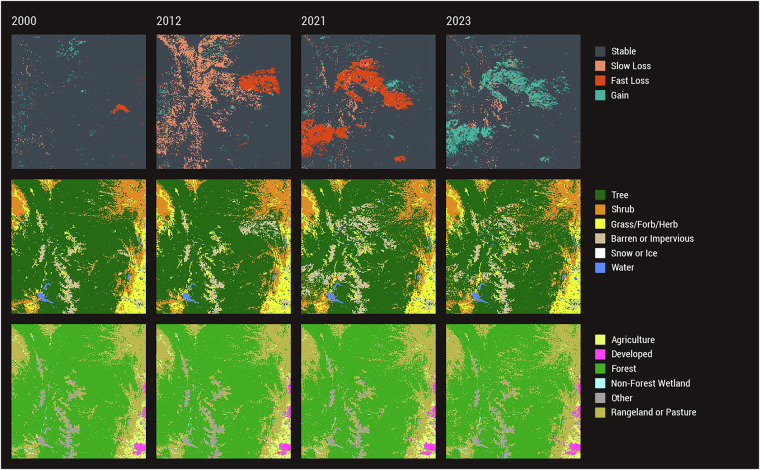
LCMS Product Suite Example – Colorado Front Range. Landscape Change Monitoring System (LCMS) Level 3 Change, Land Cover, and Land Use along Colorado’s Front Range. Slow Loss due to Mountain Pine Beetle (2012) and Fast Loss due to the Bobcat Gulch fire (2000), High Park fire (2012), Cameron Peak fire (2020), and East Troublesome fire (2020), along with vegetation cover Gain (2023) following 2020 wildfires, are illustrated. A noticeable reduction in Tree Land Cover following 2020 fires is also evident. Land Use remains largely unchanged, with only some areas converting from Forest to Rangeland or Pasture Land Use.

Figures 7–9 depict diverse stories of LCMS Change, Land Cover, and Land Use across CONUS for different event types. The Great Salt Lake, in Utah (Fig. 7), has been shrinking since 1986, reaching its historic low in 2022^62^, before rebounding slightly in 2023. The fluctuating lake shoreline is especially evident in 2023 where a large area in the northern part of the lake was covered by water once again. While the Land Cover changes rather quickly from year to year, notice that the Land Use around the lake largely remains unchanged. Conversion of dry playa to suitable rangeland can take time. During the relatively short period much of the area was converted from Water to Barren or Impervious Land Cover, the Land Use remained Other.

Hurricane Michael made landfall in the Florida panhandle in October 2018 as a Category 5 storm. Figure 8 illustrates the LCMS outputs near Pensacola, Florida before, immediately after, and several years after Hurricane Michael. Large areas of Tree Land Cover (2017) converted to Grass/Forb/Herb after the storm (2019, 2023) and the large areas that saw Fast Loss immediately following the storm (2019) experienced vegetation Gain in subsequent years (2023). While there was a lot of Loss, Gain, and change in Land Cover, Land Use remained largely unchanged. These data illustrate the use of LCMS for storm impact and recovery monitoring.

Colorado’s Front Range has experienced numerous large wildfires and extensive Mountain Pine Beetle (*Dendroctonus ponderosae*) outbreaks in recent years^63^. Figure 9 illustrates several years of landscape change impacted by these events. In 2000, Fast Loss resulting from the Bobcat Gulch fire west of Loveland, Colorado is illustrated. In 2012, large areas of Slow Loss due to Mountain Pine Beetle outbreaks and the Fast Loss due to the High Park fire west of Fort Collins are shown. The Cameron Peak and East Troublesome fires occurred in 2020, leading to large areas of Fast Loss showing up in 2021, along with the subsequent vegetation cover Gain in 2023. A noticeable conversion of Tree to Barren or Impervious Land Cover in 2021 and 2023 following the massive fires of 2020 is also evident.

These examples highlight how LCMS products relate to each other for several types of landscape change events. Some other notable examples where the suite of vegetation cover Change, Land Cover, and Land Use can provide a flexible monitoring framework may include, but are not limited to:

- Land Use conversion from Forest or Agriculture to Developed
- Forest Land Use remaining Forest after a Fast Loss event for deforestation monitoring
- Conversion of Grass/Forb/Herb to Tree over Rangeland
- Wildfire risk where there was Slow Loss over Tree areas that have yet to result in Fast Loss or Gain
- Land Use conversion from Forest to Agriculture following Fast Loss events
- Post-disturbance monitoring for areas of limited recovery

### Exploring outputs

**Fig. 10:**
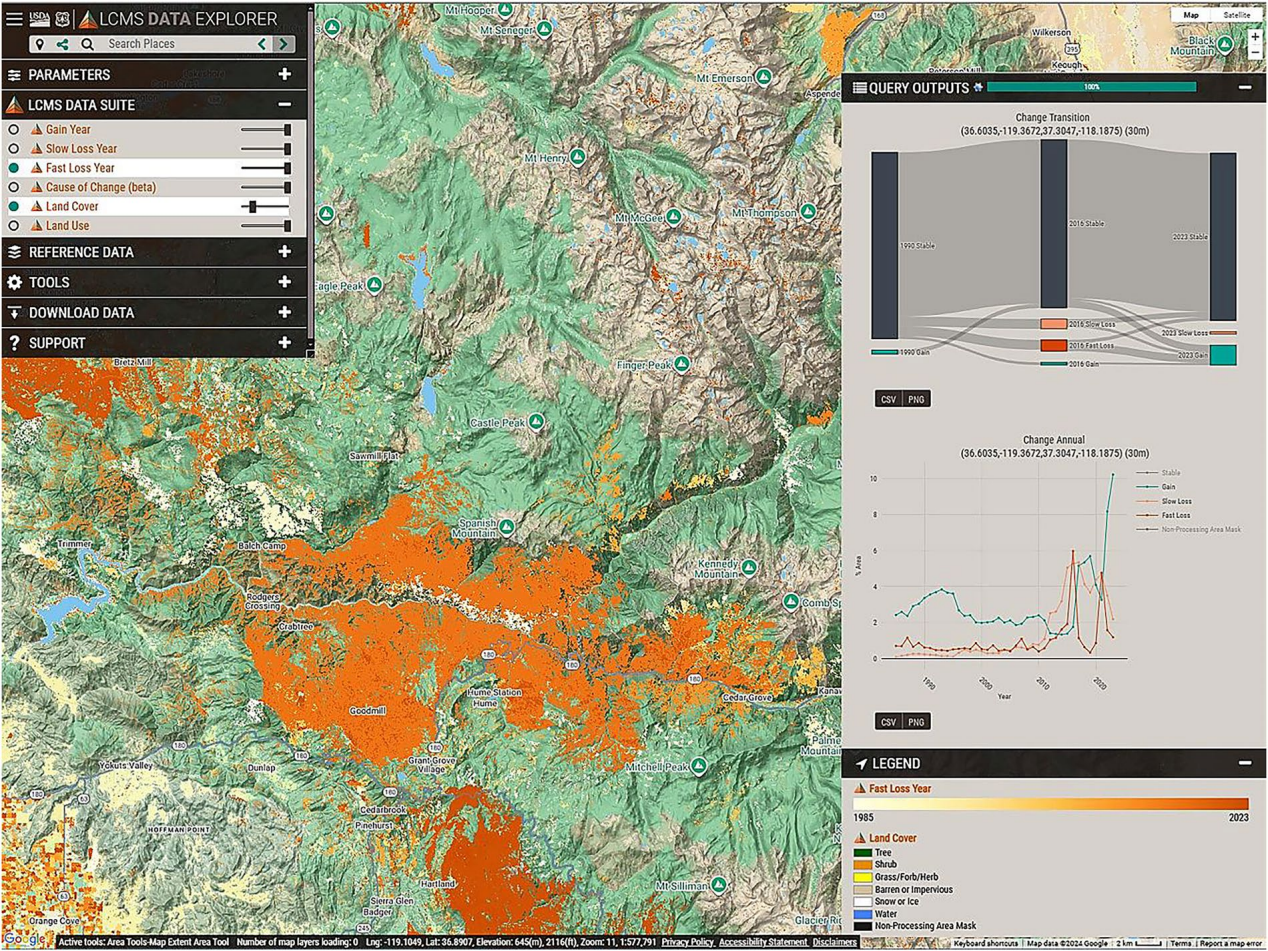
LCMS Data Explorer Example. The Landscape Change Monitoring System (LCMS) Data Explorer provides an interactive environment for exploring LCMS data. This tool can support basic visualization of LCMS data, as well as complex custom summaries.

Visualizing, summarizing, and filtering a time series map output such as LCMS can be challenging. We provide several online tools to enhance the useability of LCMS. The LCMS Data Explorer (https://apps.fs.usda.gov/lcms-viewer; Fig. 10) provides users the ability to explore the LCMS dataset in a flexible, highly interactive environment. Users can view the data on a map as individual layers or an interactive animated time lapse. They can also inspect values and summarize outputs over areas of interest. The LCMS Dashboard (https://apps.fs.usda.gov/lcms) provides quick summary reports for pre-defined summary areas such as counties and states and Forest Service Forest and District boundaries. LCMS-in-Motion (https://apps.fs.usda.gov/lcms-viewer/lcms-in-motion.html) provides an animated GIF for every National Forest and Ranger District.

### Using LCMS with FIA Data

**Fig. 11:**
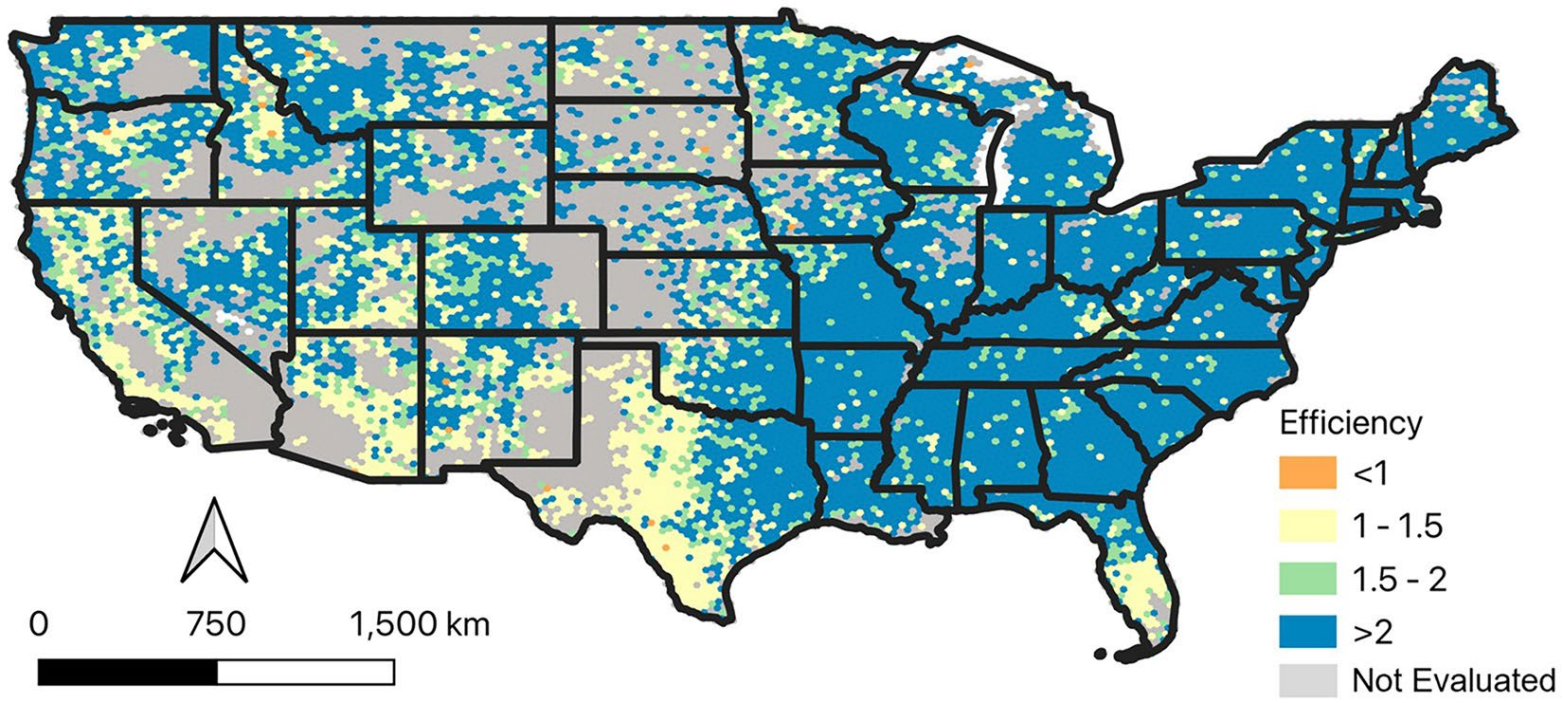
Map of Increased Efficiency of FIA Estimation Using LCMS Land Use Data. Relative efficiency of model-assisted estimation using Landscape Change Monitoring System (LCMS) Land Use Level 1 (simplified to forest/ nonforest) as ancillary data, relative to a Horvitz-Thompson estimate using the plots alone. Results are displayed by 640 km^2^ hexagons that contain an average of 27 FIA plots at the Program’s base sample intensity^67^. Analysis was performed using the MASE package^64^ in R, with hexagon membership determined by public coordinates.

As mentioned earlier, land use and land cover maps allow for more precise and more local statistical estimates when used to augment operational field inventories. A distinguishing feature of the LCMS product suite is differentiation of land use and land cover. The Land Use product is particularly promising for estimating area of forestland, a keystone application for FIA, because areas managed as forests may or may not have tree cover. When used with FIA’s field data and a generalized regression estimator (GREG) in a model-assisted context^64^, the LCMS land use map greatly improved precision of forestland area estimates within 640 km^2^ hexagons covering the US. Figure 11 displays the relative efficiency of model-assisted estimation of forestland estimates across the country. The average relative efficiency using LCMS Land Use in this context was over 3.0 per hex, meaning that three times the standard density of FIA plots would be needed to achieve the precision of model-assisted estimation with LCMS. The average relative efficiency using the LCMAP^1^ base map product, which does not differentiate use and cover, was approximately 1.25. While precision gains would vary at different scales, this use case illustrates how LCMS might practically and cost-effectively multiply the statistical power of FIA’s existing sample.

## Technical Validation

To assess final map accuracy, we use the hyperparameters and thresholds chosen in the model tuning step in a grouped, stratified, 10-fold cross-validation following Olofsson^15^ and Stehman^65^ for each Change, Land Cover, and Land Use map for each study area. We use the stratified random sample of 30 m by 30 m plot locations for each year TimeSync calibration data are available as the sample and group them by their Plot ID so that all annual samples from the same plot (that occurred in different years) are always included in the same fold (not doing so would inflate accuracy since samples from the same plot location would be in different folds and thus used for both calibration and validation). Since we use a disproportionate stratified random sample design, inverse probability sample weights are included for each sample to reduce bias introduced by over/under sampling strata. For a given stratum, this weight is the proportion of the population for that stratum, divided by the proportion of total unique sample locations for that stratum (Table 1 – 1/Weight). For example, if a plot had annual Change, Land Cover, and Land Use classes as: Stable, Tree, Forest from 1985–2000; Fast Loss, Barren or Impervious, Forest in 2001; Gain, Barren or Impervious, Forest 2002–2003; Gain, Barren and Grass/Forb/Herb Mix, Forest 2004–2007; Gain, Grass/Forb/Herb and Shrub Mix, Forest 2008–2011, Gain, Shrub, Forest 2012–2014; Gain, Shrub and Tree Mix Tree, Forest 2015–2019, all 35 years would be grouped into a single fold. They would all have the same weight since they all belong to the same stratum. The rules implemented in the map assembly are mirrored in the accuracy assessment to ensure we are assessing the accuracy of LCMS’ final maps, rather than its models.

Overall accuracy and balanced accuracy with their respective standard errors, and kappa metrics are shown for all study areas in Table 10 User’s and Producer’s accuracies for Change, Land Cover, and Land Use are shown in Tables 11–13 respectively^15^. This indicates, for example, at a 95% confidence interval, CONUS Change Level 3 has an overall accuracy of 93.32% ± 0.04*1.96 or 93.32 ± 0.078%. As the accuracy and/or weighted sum decrease, the SE will increase—notice some individual classes in Tables 11–13 have large SEs. This indicates that the precision of the accuracy of some rare and/or less accurate classes remains low. Interpretting an acceptable level of accuracy and precision depends on the application of the data.

**Table 10:** Accuracy metrics for Landscape Change Monitoring System (LCMS) products by study area.

Study Area	Product	Level	Overall Accuracy (%)	Standard Error (SE) Overall Accuracy (+/− %)	Balanced Accuracy (%)	Standard Error (SE) Balanced Accuracy (+/− %)	Kappa
CONUS	Change	1	99.1	0.02	70.98	0.54	0.53
	Change	2	93.36	0.04	61.88	0.49	0.47
	Change	3	93.32	0.04	52.5	0.75	0.46
	Land cover	1	95.78	0.03	82.63	0.25	0.6
	Land cover	2	87.25	0.06	81.21	0.21	0.76
	Land cover	3	79.02	0.07	72.22	3.22	0.69
	Land cover	4	67.03	0.08	40.46	2.28	0.57
	Land use	1	91.65	0.05	86.28	0.11	0.77
	Land use	2	84	0.06	75.76	0.26	0.77
	Land use	3	83.77	0.06	71.71	0.37	0.77
SEAK	Change	1	99.81	0.03	65.96	4.45	0.46
	Change	2	98.82	0.07	64.28	4.03	0.57
	Change	3	98.81	0.07	52.34	6.13	0.57
	Land cover	1	95.26	0.15	88.74	0.6	0.76
	Land cover	2	83.81	0.25	75.82	0.68	0.63
	Land cover	3	82.25	0.26	72.01	0.78	0.76
	Land cover	4	70.52	0.31	33.58	0.72	0.63
	Land use	1	94.04	0.17	88.56	0.46	0.8
	Land use	2	82.54	0.27	59.78	1.22	0.69
	Land use	3	86.61	0.23	68.17	2.36	0.8
PRUSVI	Change	1	96.36	0.13	67.45	1.07	0.47
	Change	2,3	77.08	0.29	53	1.03	0.28
	Land cover	1	95.26	0.15	88.74	0.6	0.76
	Land cover	2	83.81	0.25	75.82	0.68	0.63
	Land cover	3	80.38	0.27	61.14	1.7	0.57
	Land cover	4	71.34	0.31	29.21	1.2	0.48
	Land use	1	94.04	0.17	88.56	0.46	0.8
	Land use	2	82.54	0.27	59.78	1.22	0.69
	Land use	NA	82.46	0.27	56.88	1.77	0.69
HI	Change	1	95.88	0.13	61.2	1.09	0.27
	Change	2	86.89	0.23	52.09	1.24	0.27
	Change	3	86.75	0.23	40.03	1.66	0.26
	Land cover	1	95.73	0.14	95.68	0.21	0.9
	Land cover	2	86.8	0.23	86.87	0.39	0.8
	Land cover	3	79.94	0.27	78.12	0.54	0.74
	Land cover	4	74.94	0.29	36.45	0.46	0.69
	Land use	1	95.87	0.13	62.93	0.97	0.36
	Land use	2	83.88	0.25	61.14	1.19	0.76
	Land use	NA	83.88	0.25	51.07	1.09	0.76

Note, PRUSVI Change Levels 2 and 3 are the same since there is no slow loss to bin with fast loss.

**Table 11:** User’s (1−omission; precision) and producer’s (1−commission; recall) accuracies and standard errors for Change for each study area.

		User’s Accuracy (%)	User’s SE (+/− %)	Producer’s Accuracy (%)	Producer’s SE (+/− %)			User’s Accuracy (%)	User’s SE (+/− %)	Producer’s Accuracy (%)	Producer’s SE (+/− %)
**Change – Level 1**						**Change – Level 1**					
CONUS	Not-Disturbed	99.3	0.01	99.79	0.01	SEAK	Not-Disturbed	99.83	0.03	99.98	0.01
	Disturbed	71.45	0.92	42.16	0.77		Disturbed	82.85	8.2	31.93	6.3
PRUSVI	Not-Disturbed	96.84	0.12	99.42	0.05	HI	Not-Disturbed	97.27	0.11	98.5	0.08
	Disturbed	75.37	1.98	35.49	1.51		Disturbed	36.66	2.16	23.91	1.54
**Change – Level 2**						**Change – Level 2**					
CONUS	Stable	96.01	0.03	96.99	0.03	SEAK	Stable	99.38	0.05	99.43	0.05
	Loss	42.49	0.77	42.16	0.77		Loss	42.97	7.76	31.93	6.3
	Gain	55.3	0.38	46.49	0.35		Gain	60.55	3.02	61.48	3.03
PRUSVI	Stable	87.03	0.26	86.15	0.26	HI	Stable	94.1	0.17	91.91	0.19
	Loss	39.05	1.62	35.49	1.51		Loss	23.42	1.52	23.91	1.54
	Gain	34.19	0.85	37.36	0.91		Gain	28.85	1.15	40.45	1.47
**Change – Level 3**						**Change – Level 3**					
CONUS	Stable	96	0.03	96.99	0.03	SEAK	Stable	99.38	0.05	99.43	0.05
	Slow Loss	22.97	1.31	19.21	1.12		Slow Loss	19.8	14.39	12.27	9.32
	Fast Loss	45.31	0.91	47.32	0.93		Fast Loss	46.81	8.71	36.18	7.37
	Gain	55.27	0.38	47.32	0.35		Gain	60.52	3.02	61.48	3.03
PRUSVI	Stable	87.03	0.26	86.15	0.26	HI	Stable	94.06	0.17	91.91	0.19
	Slow Loss	Not Modeled					Slow Loss	20.25	1.49	21.77	1.59
	Fast Loss	39.05	1.62	35.49	1.51		Fast Loss	11.98	4.87	5.98	2.51
	Gain	34.19	0.85	37.36	0.91		Gain	28.83	1.15	40.45	1.47

**Table 12:** User’s (1−omission; precision) and producer’s (1−commission; recall) accuracies and standard errors for Land Cover for each study area.

		User’s Accuracy (%)	User’s SE (+/− %)	Producer’s Accuracy (%)	Producer’s SE (+/− %)			User’s Accuracy (%)	User’s SE (+/− %)	Producer’s Accuracy (%)	Producer’s SE (+/− %)
**Land Cover – Level 1**						**Land Cover – Level 1**					
CONUS	Vegetated	98.23	0.02	97.3	0.03	SEAK	Vegetated	98.59	0.1	94.7	0.18
	Non-Vegetated	57.91	0.34	67.95	0.35		Non-Vegetated	88.86	0.37	96.9	0.21
PRUSVI	Vegetated	97.66	0.11	97.02	0.12	HI	Vegetated	97.8	0.12	95.84	0.16
	Non-Vegetated	76.22	0.88	80.45	0.84		Non-Vegetated	91.68	0.32	95.52	0.25
**Land Cover – Level 2**						**Land Cover – Level 2**					
CONUS	Tree-Veg	90.29	0.08	85.25	0.1	SEAK	Tree-Veg	84.74	0.39	86.79	0.37
	Non-Tree Veg	88.4	0.07	90.44	0.07		Non-Tree Veg	81.22	0.49	71.95	0.53
	Non-Veg	57.91	0.34	67.95	0.35		Non-Veg	88.86	0.37	96.9	0.21
PRUSVI	Tree-Veg	88.14	0.26	92.31	0.22	HI	Tree-Veg	87.15	0.39	85.83	0.4
	Non-Tree Veg	68.64	0.82	54.7	0.78		Non-Tree Veg	81.3	0.47	79.25	0.48
	Non-Veg	76.22	0.88	80.45	0.84		Non-Veg	91.68	0.32	95.52	0.25
**Land Cover – Level 3**						**Land Cover – Level 3**					
CONUS	Tree	90.29	0.08	85.25	0.1	SEAK	Tree	87.15	0.37	86.81	0.37
	Shrub	74.66	0.17	59.65	0.17		Shrub	78.22	0.52	78.55	0.52
	Grass	73.81	0.12	87.21	0.1		Grass	16.5	2.73	3.87	0.69
	Barren	40.8	0.41	56.2	0.49		Barren	57.05	1.08	82.78	0.99
	Snow	86.4	6.53	62.86	7.85		Snow	92.06	0.4	91.35	0.41
	Water	93.36	0.31	82.15	0.45		Water	90.02	1.21	88.68	1.27
PRUSVI	Tree	88.14	0.26	92.31	0.22	HI	Tree	87.15	0.39	85.83	0.4
	Shrub	42.09	2.69	8.08	0.65		Shrub	55.75	1.12	34.08	0.84
	Grass	47.29	0.93	59.63	1.03		Grass	61.62	0.68	77.96	0.66
	Barren	74.68	0.92	81.21	0.87		Barren	86.86	0.49	92.75	0.39
	Water	90.74	2.5	64.47	3.49		Water	100	0	100	0
**Land Cover – Level 4**						**Land Cover – Level 4**					
CONUS	Tree	81.48	0.12	93.79	0.08	SEAK	Tree	81.76	0.44	91.95	0.33
	Tall Shrb-Tre	Not Modeled					Tall Shrb-Tre	0	0	0	0
	Shrub-Tree	21.09	1.61	2.19	0.19		Shrub-Tree	15.48	1.78	7.38	0.89
	Grass-Tree	37.48	0.44	16.88	0.23		Grass-Tree	7.77	11.8	0.4	0.63
	Barren-Tree	4.12	1.21	0.45	0.23		Barren-Tree	40	78.99	0.36	0.9
	Tall Shrub	Not Modeled					Tall Shrub	61.25	0.74	76.83	0.72
	Shrub	40.02	0.41	20.12	0.24		Shrub	24.8	1.03	23.6	0.99
	Grass-Shrb	37.89	0.24	40.65	0.25		Grass-Shrb	17.88	2.49	5.93	0.88
	Barren-Shrb	33.32	0.52	21.22	0.36		Barren-Shrb	0	0	0	0
	Grass	73.34	0.12	89.01	0.1		Grass	3.67	1.53	0.82	0.35
	Barren-Gra	0.64	0.58	0.04	0.04		Barren-Gra	0	0	0	0
	Barren	40.8	0.41	56.2	0.49		Barren	57.05	1.08	82.78	0.99
	Snow	86.4	6.53	62.86	7.85		Snow	92.06	0.4	91.35	0.41
	Water	93.36	0.31	82.15	0.45		Water	90.02	1.21	88.68	1.27
PRUSVI	Tree	76.87	0.34	95.92	0.18	HI	Tree	80.76	0.46	94.42	0.29
	Tall Shrb-Tre	Not Modeled					Tall Shrb-Tre	Not Modeled			
	Shrub -Tree	NA	NA	0	0		Shrub -Tree	NA	NA	0	0
	Grass-Tree	36.3	3.29	4.96	0.55		Grass-Tree	11.32	6.03	0.31	0.18
	Barren-Tree	NA	NA	0	0		Barren-Tree	NA	NA	0	0
	Tall Shrub	Not Modeled					Tall Shrub	Not Modeled			
	Shrub	46.94	3.58	12.36	1.21		Shrub	30.58	1.17	28.29	1.1
	Grass-Shrb	5.31	1.88	0.79	0.29		Grass-Shrb	16.63	1.85	5.25	0.62
	Barren-Shrb	NA	NA	0	0		Barren-Shrb	0	0	0	0
	Grass	46.36	0.93	61.63	1.05		Grass	60.88	0.69	79.98	0.65
	Barren-Gra	0	0	0	0		Barren-Gra	0	0	0	0
	Barren	74.68	0.92	81.21	0.87		Barren	86.86	0.49	92.75	0.39
	Snow	Not Modeled					Snow	Not Modeled			
	Water	90.74	2.5	64.47	3.49		Water	100	0	100	0

**Table 13:** User’s (−-omission; precision) and producer’s (1−commission; recall) accuracies and standard errors for Land Use for each study area.

		User’s Accuracy (%)	User’s SE (+/− %)	Producer’s Accuracy (%)	Producer’s SE (+/− %)			User’s Accuracy (%)	User’s SE (+/− %)	Producer’s Accuracy (%)	Producer’s SE (+/− %)
**Land Use – Level 1**						**Land Use – Level 1**					
CONUS	Anthropogenic	91.06	0.11	75.14	0.15	SEAK	Anthropogenic	96.6	2.54	50.33	5.05
	Non-Anthro	91.81	0.05	97.42	0.03		Non-Anthro	99.78	0.03	99.99	0.01
PRUSVI	Anthropogenic	89.21	0.52	79.48	0.64	HI	Anthropogenic	67.3	2.32	26.5	1.37
	Non-Anthro	95.08	0.17	97.63	0.12		Non-Anthro	96.42	0.13	99.35	0.06
**Land Use – Level 2**						**Land Use – Level 2**					
CONUS	Agriculture	91.23	0.12	75.81	0.16	SEAK	Agriculture	Not Modeled			
	Developed	75.86	0.38	60.56	0.39		Developed	96.6	2.54	50.33	5.05
	Forest	85.97	0.1	91.05	0.08		Forest	85.72	0.39	88.3	0.36
	Other	77.96	0.37	63.53	0.38		Other	94.85	0.26	87.42	0.37
	Rangeland	80.23	0.11	87.86	0.09		Rangeland	78.82	0.5	84.4	0.46
PRUSVI	Agriculture	29.4	3.36	12.28	1.56	HI	Agriculture	29.9	3.19	15.92	1.86
	Developed	90.79	0.5	86.17	0.58		Developed	93.39	1.74	29.09	1.78
	Forest	87.37	0.29	92.28	0.24		Forest	85.75	0.44	86.14	0.44
	Other	56.59	2.17	46.38	1.98		Other	92.62	0.33	88.36	0.39
	Rangeland	63.9	0.81	61.77	0.8		Rangeland	77.2	0.45	86.18	0.39
**Land Use – Level 3**						**Land Use – Level 3**					
CONUS	Agriculture	91.23	0.12	75.81	0.16	SEAK	Agriculture	Not Modeled			
	Developed	72.19	0.38	63.02	0.38		Developed	93.43	3.41	50.33	5.05
	Forest	86.22	0.1	90.43	0.09		Forest	85.72	0.39	88.28	0.36
	NFW	59.38	0.78	42.06	0.66		NFW	56.91	2.61	22.32	1.38
	Other	81.24	0.42	71.06	0.45		Other	96.45	0.22	95.52	0.25
	Rangeland	80.23	0.11	87.86	0.09		Rangeland	78.82	0.5	84.4	0.46
PRUSVI	Agriculture	29.4	3.36	12.28	1.56	HI	Agriculture	29.9	3.19	15.92	1.86
	Developed	89.72	0.51	87.23	0.56		Developed	93.39	1.74	29.09	1.78
	Forest	87.66	0.29	91.99	0.25		Forest	85.75	0.44	86.14	0.44
	NFW Wetland	36.69	2.6	36.75	2.6		NFW	NA	NA	0	0
	Other	84.69	2.71	51.28	2.92		Other	92.62	0.33	89.08	0.38
	Rangeland	63.9	0.81	61.77	0.8		Rangeland	77.2	0.45	86.18	0.39

While current map outputs are delivered at the highest level, lower levels can be achieved by cross-walking the higher-level outputs accordingly (e.g. Land Cover Shrub and Tree Mix would crosswalk to Tree (Level 3), Tree Vegetated (Level 2), and Vegetated (Level 1)).

The core focus of LCMS is monitoring vegetation cover change. Since this is a rare class, focusing on overall accuracy of any map output can be misleading. For example, the relatively high overall accuracy found in our Change output is deceivingly high. Balanced accuracy is a more appropriate representation of the overall map accuracy when rare classes, such as loss, are of interest. Despite being quite low, Change class accuracies are similar to those reported in similar studies such as Cohen *et al*.^66^ and Cohen *et al*.^43^. Also quite low, the accuracy of the Land Cover and Land Use classes are generally similar to those found in Gorelick *et al*.^12^ and Pengra *et al*.^23^.

Because TimeSync uses manual image interpretation and ancillary information, it is generally more sensitive to subtle change and presence of rare or spectrally variable classes than a model-assisted approach. Also, our mixed Land Cover Level 4 classes are inherently difficult to model accurately due to their mixed nature. When the Land Cover Level 4 classes are binned into broader groups in Land Cover Level 3, accuracies increase. Land Cover Level 3 serves as the core Land Cover level that balances thematic detail and class accuracy. We suggest using Level 3 for any application that does not need the additional information provided with Land Cover Level 4 classes. Despite their lower accuracy, the Land Cover Level 4 mixed classes are necessary for some rangeland, low magnitude disturbance, and post disturbance succession monitoring needs.

The accuracy results provide an understanding of how to properly use LCMS outputs. Many rare and/or spectrally variable Change, Land Cover, and Land Use classes should be used more conservatively to identify significant changes in the higher classification levels. Lower classification levels will coarsen the thematic detail but provide greater confidence when determining whether a change is significant. There are nearly infinite methods for cross-walking LCMS classes both within and between products. It is ultimately up to users to choose a cross-walking method that allows them to answer their question with the desired level of confidence^66^.

## Supplementary information

Supplementary Table 1

## Data Availability

LCMS data are distributed in three different locations to meet different user needs. Please refer to Table 9 for detailed data location and descriptions.

## Code availability

The full LCMS workflow is available as a training course developed as a collaboration between the Forest Service, RedCastle Resources Inc., and Google (https://github.com/redcastle-resources/lcms-training). This course allows users to reproduce LCMS outputs over the PRUSVI study area in a series of Python notebooks. It also provides users the ability to apply these methods across their own study areas.
