# Supplementary material for: Coincident maps of changing land cover, land use, and forest condition in the United States, 1985-present
Source: Sci Data. 2026 Mar 3;13:575. doi: 10.1038/s41597-026-06743-0 (PMC13065782; doi:10.1038/s41597-026-06743-0)
Supplement: Supplementary file 1 — Supplementary Table 1 [file 41597_2026_6743_MOESM1_ESM.docx]

Supplementary Table 1.–Map assembly rules and associated ancillary datasets.

|  | **Description** | **CONUS** | **SEAK** | **PRUSVI** | **HI** |
| --- | --- | --- | --- | --- | --- |
| **General Developed Land Use (LU) Probability Threshold** | Developed LU must be above a threshold or the next highest probability LU will be used | X | X | X | X |
| **Coastal Developed LU Probability Threshold** | Developed LU that is less than 6 meters (SEAK, PRUSVI, and HI) or 10 meters (CONUS) above sea level must be above a threshold or the next highest probability LU will be used | X | X | X | X |
| **General Non-Forest Wetland LU Probability Threshold** | Non-Forest Wetland LU must be above a threshold or the next highest probability LU will be used | X | X | X | X |
| **Forest and Agriculture LU Probability Threshold** | If Forest LU is above a threshold then Agriculture LU probability is set to zero and the next highest probability LU will be used | X |  |  |  |
| **CDL Agriculture LU Probability Threshold** | Agriculture LU that intersects CDL non-tree crop data must be above a threshold or the next highest probability LU will be used | X |  |  |  |
| **CDL Tree Agriculture and Agriculture LU Probability Threshold** | If Agriculture LU intersects CDL tree crop data and Tree Land Cover is above a threshold then Agriculture LU probability is set to the highest probability | X |  |  |  |
| **CDL Fallow Fields Agriculture LU Probability Threshold** | Rangeland or Pasture LU that intersects CDL fallow data must be above a threshold or the next highest probability LU will be used | X |  |  |  |
| **General Agriculture LU Probability Threshold** | Agriculture LU must be above a threshold or the next highest probability LU will be used |  |  | X | X |
| **Probability Thresholds to Prevent Forest Commission in Agriculture LU** | If Forest LU that intersects CDL non-tree crop or CDL fallow is below a threshold then Forest LU probability is set to zero |  |  |  |  |
| **Probability Thresholds to Prevent Conversion of Forest to Developed LU after Timber Harvest** | If Developed LU does not intersect the GHSL builtup layer, is below a threshold, and Rangeland or Pasture LU is above a threshold then Developed LU probability is set to zero | X |  |  |  |
| **Probability Threshold to Prevent Forest LU Commission in GHSL Builtup** | If Forest LU that intersects the GHSL builtup layer is below a threshold and developed LU is above a threshold then Forest LU probability is set to zero | X | X |  | X |
| **Probability Threshold to Prevent Forest LU Commission in Intertidal Zone** | Forest LU less than 10 meters above sea level must be above a threshold or the next highest probability LU will be used |  | X |  |  |
| **General Other LU Probability Threshold** | Other LU must be above a threshold or the next highest probability LU will be used |  |  |  | X |
| **Probability Threshold to Prevent Agriculture LU Commission in GHSL Builtup** | Agriculture LU that intersects the GHSL builtup layer must be above a threshold or the next highest probability LU will be used |  |  |  | X |
| **Elevation Threshold to Prevent Rangeland or Pasture LU Commission in Ocean** | Areas intersecting the JRC Water layer and less than 1 meter above sea level have Other LU probability set to 99 and Rangeland or Pasture LU probability set to zero |  |  |  | X |
| **Coastal Tree Land Cover (LC) Probability Threshold** | Tree LC classes less than 2 meters above sea level must be above a threshold or the next highest probability LC will be used |  | X |  |  |
| **Ocean Snow or Ice LC Probability Threshold** | Snow or Ice LC classes less than 2 meters above sea level must be above a threshold or the next highest probability LC will be used |  | X |  |  |
| **General Grass/Forb/Herb LC Probability Threshold** | Grass/Forb/Herb LC must be above a threshold or the next highest probability LC will be used |  |  |  | X |
| **Elevation and Probability Threshold to Prevent Barren or Impervious LC Commission in Ocean** | Areas intersecting the JRC Water layer and less than 1 meter above sea level and meeting a probability threshold for Water LC have Barren or Impervious LC probability set to zero and Water LC probability set to 99 |  |  |  | X |
